# Decline Variability of Cortical and Subcortical Regions in Aging: A Longitudinal Study

**DOI:** 10.3389/fnhum.2020.00363

**Published:** 2020-09-04

**Authors:** Silvano Sele, Franziskus Liem, Susan Mérillat, Lutz Jäncke

**Affiliations:** ^1^Division Neuropsychology, Department of Psychology, University of Zurich, Zurich, Switzerland; ^2^University Research Priority Program (URPP), “Dynamics of Healthy Aging”, University of Zurich, Zurich, Switzerland

**Keywords:** aging, structural MRI, latent growth curve model, longitudinal data analysis, cortical and subcortical brain structure trajectories

## Abstract

Describing the trajectories of age-related change for different brain structures has been of interest in many recent studies. However, our knowledge regarding these trajectories and their associations is still limited due to small sample sizes and low numbers of repeated measures. For the present study, we used a large longitudinal dataset (four measurements over 4 years) comprising anatomical data from a sample of healthy older adults (*N* = 231 at baseline). This dataset enables us to gain new insights about volumetric cortical and subcortical changes and their associations in the context of healthy aging. Brain structure volumes were derived from T1-weighted MRI scans using FreeSurfer segmentation tools. Brain structure trajectories were fitted using mixed models and latent growth curve models to gain information about the mean extent and variability of decline trajectories for different brain structures as well as the associations between individual trajectories. On the group level, our analyses indicate similar linear changes for frontal and parietal brain regions, while medial temporal regions showed an accelerated decline with advancing age. Regarding subcortical regions, some structures showed strong declines (e.g., hippocampus), others showed little decline (e.g., pallidum). Our data provide little evidence for sex differences regarding the aforementioned trajectories. Between-person variability of the person-specific slopes (random slopes) was largest in subcortical and medial temporal brain structures. When looking at the associations between the random slopes from each brain structure, we found that the decline is largely homogenous across the majority of cortical brain structures. In subcortical and medial temporal brain structures, however, more heterogeneity of the decline was observed, meaning that the extent of the decline in one structure is less predictive of the decline in another structure. Taken together, our study contributes to enhancing our understanding of structural brain aging by demonstrating (1) that average volumetric change differs across the brain and (2) that there are regional differences with respect to between-person variability in the slopes. Moreover, our data suggest (3) that random slopes are highly correlated across large parts of the cerebral cortex but (4) that some brain regions (i.e., medial temporal regions) deviate from this homogeneity.

## Introduction

Growing old is accompanied by physical and cognitive declines. It is of great importance to know how these declines can be kept at a minimum so that the quality of life can remain high. By using brain imaging techniques like magnetic resonance imaging (MRI), one can observe that the brain does undergo pronounced changes with aging (Oschwald et al., 2019). Many recent studies have described aging-related change for different brain structures (Du et al., 2006; Leonard et al., 2008; Driscoll et al., 2009; Fjell and Walhovd, 2010; Raz et al., 2010, 2005; Walhovd et al., 2011; Ziegler et al., 2012; Fjell et al., 2013, 2009; Pfefferbaum et al., 2013; Storsve et al., 2014; Fraser et al., 2015; Jäncke et al., 2015; Potvin et al., 2016; Coupé et al., 2017; Narvacan et al., 2017; Vinke et al., 2018). For most brain structures investigated in these studies, an average annual volumetric decline ranging between 0.2 and 0.8% has been reported. This volumetric decline comes with some regional variability, meaning that some brain structures are more sensitive to age decline than others, with no clear consensus about which brain region or cortical lobe shows the largest volumetric losses. Furthermore, the results of these studies were heterogeneous, whether the aging-related changes differ between men and women. Besides the extent of decline, many of the aforementioned studies were also interested in whether the trajectories of volumetric decline follow linear or non-linear shapes. Accelerated decline with advancing age has repeatedly been reported in some subcortical and medial temporal lobe structures, while the decline of total gray matter volume has been reported to follow a more linear shape (e.g., Walhovd et al., 2011; Ziegler et al., 2012; Vinke et al., 2018). In general, however, the decline patterns of subcortical structures in the last decades of life seem to be rather diverse (e.g., Walhovd et al., 2011; Ziegler et al., 2012). Generally, estimates of average trajectories seem to be quite noisy because information about average annual decline rates often comes from suboptimal sources, such as (a) cross-sectional studies (e.g., Fjell and Walhovd, 2010; Walhovd et al., 2011; Ziegler et al., 2012), (b) longitudinal studies with only two repeated measures (e.g., Raz et al., 2005; Fjell et al., 2009; Storsve et al., 2014; Narvacan et al., 2017), or (3) longitudinal studies that did not separate the between-subject from the within-subject effects by using between-subject information in estimation of average trajectories (e.g., Driscoll et al., 2009; Narvacan et al., 2017; Vinke et al., 2018). Estimation of average trajectories from cross-sectional data is very noisy because there is a lot of between-subject variance in these brain structures. In addition, the estimates may be more easily biased by other factors (e.g., cohort effects). Secondly, because longitudinal MRI studies are resource-intensive, the number of repeated measures usually falls short. This is problematic because three repeated measures are minimally required to properly estimate a linear slope model with random intercepts and random slopes, while four or more repeated measures are preferred (Muthén and Curran, 1997; Curran et al., 2010). Although there was already a body of research dedicated to structural brain changes in aging, data from longitudinal studies with more than three repeated measures would be beneficial for more accurate estimation. Lastly, by separating between-subject from within-subject variances, longitudinal studies result in more precise estimates for longitudinal changes.

Most of the previous studies (Du et al., 2006; Leonard et al., 2008; Driscoll et al., 2009; Fjell and Walhovd, 2010; Walhovd et al., 2011; Ziegler et al., 2012; Fjell et al., 2013, 2009; Pfefferbaum et al., 2013; Storsve et al., 2014; Fraser et al., 2015; Jäncke et al., 2015; Potvin et al., 2016; Coupé et al., 2017; Narvacan et al., 2017; Vinke et al., 2018) focused on population average annual structural brain changes. While these average structural changes surely are of interest, they provide only a part of the characterization of aging-related change because aging affects people differently. Consequently, we would expect that the changes in brain structure also vary between individuals, even in healthy aging people. There may be brain structures that show high variability in the amount of decline between individuals, while others may show only marginal variability. Brain structures that show high between-person variability are of special interest because these structures may be more vulnerable to environmental factors and lifestyle choices (Raz et al., 2005). In addition, estimating the associations between the person-specific brain structure trajectories would provide important information about the heterogeneity of the aging process in the brain. By this, we could investigate if a person with a faster-than-average decline in brain structure A also has a faster-than-average decline in brain structure B. Associations between the trajectories of two brain structures can be classified into level-level, level-change, change-change associations (Oschwald et al., 2019). A level-level association represents the covariance parameter between random intercepts, level-change represents the covariance between random intercepts and random slopes, and change-change represents the covariance between random slopes. So far, slope variability of different brain structures and slope associations between brain structures have only been described in the study by Raz et al. (2005). The authors reported similar random slope variability for almost all of the brain structures they studied. Evidence for an association, however, was only observed for about one-quarter of the studied slope-to-slope associations (Raz et al., 2005). In a later study, Raz et al. (2010) confirmed their previous results regarding slope variability but did not report any associations between the trajectories (Raz et al., 2010, 2005). Due to the limited number of repeated measures, small sample sizes in combination with wide age ranges in these two studies, the resulting estimates should be taken with caution.

Using a longitudinal dataset with four measurement occasions over the span of 4 years, our main goal therefore was (1) to provide more information on the average volumetric decline of lobular and subcortical structures as well as its shape (acceleration of decline with advancing age) and predictors (e.g., potential sex differences) and, most importantly, (2) to gain new insights about the variations of and the associations between person-specific brain structure trajectories in healthy aging.

## Materials and Methods

### Sample Description

Structural MRI data were taken from the Longitudinal Healthy Aging Brain Database Project (LHAB; Switzerland) – an ongoing project conducted at the University of Zurich (Zöllig et al., 2011). We used data from the first four measurement occasions (baseline, 1-year follow-up, 2-year follow-up, 4-year follow-up). For 24 subjects, additional 3-year follow-up data were collected. The baseline LHAB dataset included 232 participants, of which 231 had MRI data and were therefore included in the current analysis (age at baseline: *M* = 70.8, range = 64–87; females: 113). At each measurement occasion, participants completed an extensive battery of neuropsychological and psychometric cognitive tests and underwent brain imaging. The brain imaging data was usually acquired in the same week as the behavioral assessments. Inclusion criteria for study participation at baseline were age ≥64, right-handedness, fluent German language proficiency, a score of ≥26 on the Mini Mental State Examination (MMSE; Folstein et al., 1975), no self-reported neurological disease of the central nervous system and no contraindications to MRI. Participation was voluntary and all participants gave written informed consent in accordance with the declaration of Helsinki. Self-reported physical and mental health of the sample at baseline, as measured by the SF-12 (Ware et al., 1996), were 50.9 ± 7.4 (M ± SD) and 54.8 ± 6.3, respectively, which indicates above-average health compared to a norm population (Ware et al., 1995). As expected, sample means for these general health indicators slightly declined over time, but still indicated above-average health at 4-year follow-up (physical health score: 50.5 ± 6.9, mental health score: 53.1 ± 8.0, MMSE = 28.3 ± 1.3). At 4-year follow-up, the structural MRI dataset still comprised 72% of the baseline sample (*N* = 166). The exact attrition pattern is shown in Supplementary Table S1. We were assuming that the missing mechanism was missing at random, meaning that - given the covariates and observed values – missingness should not depend on unobserved values (Bhaskaran and Smeeth, 2014). By using the normalization of the age category of 70–90 years for the entire sample, the mean IQ of the sample was 120.6 (SD = 6.7) at baseline. In follow-up measurements, the participants achieved on average similar IQ scores (Jäncke et al., 2019). Finally, acquisition and processing of MRI data is prone to unwanted influences and errors. We excluded subjects who had rather large influence on parameter estimation as indicated by the Cook’s distance and the loglikelihood contribution of observations (Cook and Weisberg, 1982; Cook, 1986). These Influence measures were obtained with latent growth curve models using the time window approach for the time intervals. Subjects having a Cook’s distance > 0.5 and a likelihood contribution of < −7.5 or a Cook’s distance > 1 and a likelihood contribution of < −4 were excluded. These values were chosen based on visual inspection of the excluded subjects. Depending on the brain structure, 0 to 4 subjects were excluded. The LHAB sample has been used in previous publications of our group (e.g., Jäncke et al., 2019; Oschwald et al., 2019).

### Image Acquisition

Magnetic resonance imaging scanning was carried out at the University Hospital of Zurich on a 3.0T Philips Ingenia scanner (Philips Medical Systems, Best, Netherlands). T1-weighted images were recorded with a gradient echo sequence (3D turbo field echo, 160 sagittal slices, slice thickness = 1 mm, in-plane resolution = 1 × 1 mm, FOV = 240 × 240 mm, repetition time = 8.18 ms, echo time = 3.80 ms, flip angle = 8).

### Image Processing

FreeSurfer (v5.3, Fischl, 2012) as implemented in the FreeSurfer BIDS-App (Gorgolewski et al., 2017) was used to obtain volumetric measurements of cortical and subcortical structures using the Desikan-Killiany parcellation scheme (Desikan et al., 2006). As part of our data processing pipeline, the structural MR images were visually inspected for good SNR and obvious artifacts (such as motion). In addition, the surfaces, created by the FreeSurfer software, were carefully visually checked for gross deviations. Only very few images (*N* = 24) had to be excluded from the sample due to insufficient data quality. In addition, for some images (chosen at random), the surfaces, created by the FreeSurfer software, were visually checked for gross deviations.

We deliberately refrained from applying manual correction of the reconstructed surfaces given that previous research showed limited applicability of manual corrections (McCarthy et al., 2015). Furthermore, we believe that manual corrections, particularly in case of longitudinal structural MRI data, can bias the images, not only through between-rater discrepancies but also through inconsistencies in applying corrections across time points.

The sum of the left and the right hemisphere volumes were used for each brain structure. We used the sum of the left and the right hemisphere volumes because we think that the noise in volumetric MRI data is rather high to quantify more fine-grained changes between the hemispheres. Over the chosen brain parcellation, we expect the slopes between the hemispheres to be highly correlated. Furthermore, by using bi-lateral sums over both hemispheres, we could reduce the number of variables. Fewer variables facilitates visualization and pattern detection. Likewise, to keep the number of variables low, we decided to focus on volumetric changes, as one of the most important and widely used structural measures. Additionally, the sole focus on volumetric changes in cortical regions facilitates the comparison with changes seen in subcortical regions.

### Statistical Modeling

The trajectories of the brain structures were fitted with linear random slope models, allowing for person-specific intercepts and slopes. To separate the between-subject variance from the within-subject variance, the age predictor was separated in the age-at-study-entry predictor (entry-age) and in the time-difference-between-the-baseline-and-the-subsequent-measurements predictor (slope) (Grimm et al., 2016). Because there were some small deviations from the planned time interval between measures for some subjects, we used the exact follow-up time in the mixed model framework and used a time-window approach in the latent growth curve framework (Grimm et al., 2016). The models were slightly modified depending on the predictors and parameters of interest as described below.

Average population trajectories were fitted with linear random slope models, which included as predictors: slope, intracranial volume (ICV), entry-age, (entry-age)^2^, slope × entry-age, and slope × ICV interaction. Main parameters of interest were the slope and the slope × entry-age interaction. These two parameters determine the average volumetric decline. The slope × entry-age interaction indicates whether a larger decline with advancing age at study entry is expected. The expected volumetric loss over the span of 20 years (starting at age 65 with an updated slope every 4 years until age 85) was extrapolated from the linear combination of the slope and the slope × entry-age interaction parameter. Bootstrap was used to gain information about the variability of these estimates, as well as about fastest declining brain structures by comparing the extrapolated volumetric declines between each brain structure to each other brain structure in a pairwise fashion. A bootstrapped mass of Region A > Region B was obtained by calculating the percent of bootstrap samples where the decline in region A was estimated to be larger than the decline in region B.

To investigate the effect of sex, average trajectories were fitted for men and women using the same basic model as described above with additional predictors for sex, sex × entry-age, (sex × entry-age)^2^, sex × slope, and sex × slope × entry-age interactions. Main parameters of interest were the sex × slope and sex × slope × entry-age interactions, as well as the random slope variance parameter. The sex × slope and sex × slope × entry-age interactions quantify the difference between the slopes of men and the slopes of women. The sex × slope × entry-age interaction parameter provides information whether one of the sexes declines faster with advancing age. The random slope variance parameter quantifies how much individuals deviate from the average slopes and therefore shows how heterogenous the decline is in the population.

To evaluate how the decline of brain structures is related to changes in other brain structures, we fitted associations between the trajectories of the different brain structures with bivariate growth models. We applied basically the same (univariate) model as described in the last paragraph but allowing for associations between random intercepts, random slopes, and within-subject errors (at the same time point) between the models. In this analysis, we were mainly interested in the correlation between random slopes. Consequently, only these correlations are reported in the corresponding results section. The results of other analyzed types of associations are provided in the Supplementary Figures S8, S9.

**FIGURE 1:**
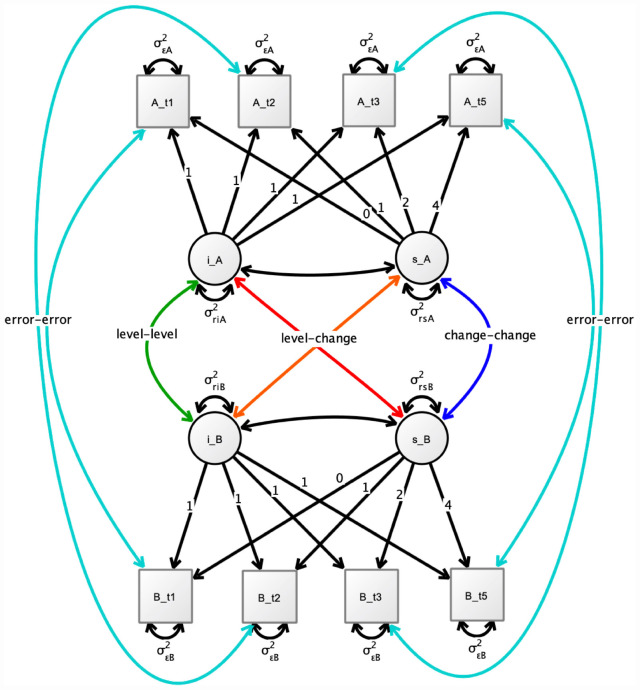
Simplified illustration of the used bivariate growth model for two brain structures A and B. i_ = random intercept. s_ = random slope. Not shown are the included covariates on the intercept and slopes. Also, the separation of the time intervals into eleven time bins from 0 to 4.5 years is not illustrated. The main interest in our study was on the change-change associations between brain structures. t1: baseline, t2 = 1-year follow-up, t3 = 2-year follow-up, 5 = 4-year follow-up.

We used a time window approach to approximate the exact time difference between measurements. The number of time bins and the distances were chosen with a k-median algorithm implemented in the R package *Ckmeans.1d.dp* (Wang and Song, 2011). This resulted in eleven time-bins (0, 1.0, 1.1, 2.0, 2.1, 2.2, 3.0, 4.0, 4.1, 4.2, 4.5 years). Models were estimated using Bayesian estimation with the default priors implemented in Mplus [prior for variance-covariance matrices of size p ∼ Inverse-Wishart (0, -p-1), priors for intercepts and slopes ∼ Normal (0,10^10^)]. This default covariance prior corresponds to a uniform prior on (-infinity to infinity) for all elements of the covariance matrix (Asparouhov and Muthén, 2010a). A graphical representation of the fitted bivariate models is shown in Figure 1. Bayesian estimation was used (instead of maximum likelihood) because the estimated random slope variance parameters were very small (compared to the within-subject error) for some brain structures. The small random slope parameters may have led to estimation difficulties in the maximum likelihood framework.

To obtain a simple estimate of the multivariate correlation structure on which it was possible to do principal component analysis (PCA), we sampled the plausible values (Asparouhov and Muthén, 2010b) of the random slopes from each of the bivariate models, connecting each structure to each other structure. To obtain just one random slope estimate per person for each structure, we used the mean of the means from the distributions of the plausible values of the bivariate models. The random slope estimates were adjusted for the covariates ICV, sex, and entry-age using regression. Note that this simple estimate will be biased and may underestimate or overestimate the correlations. However, the general covariance pattern should be retained. Visualizing the principal components may reveal some patterns that might not be apparent by looking at the bivariate correlations.

### Data Scaling

The various brain regions differ in their total size. To put the amount of volumetric loss on comparable scales between the different brain structures, each brain structure was divided by its intercept multiplied by 100 (at an age of 65 years estimated with linear random slope models including as covariates on the intercept: ICV (grand mean-centered), entry-age, (entry-age)^2^. Using this scaling, the slope parameter corresponds to the annual percent change of the intercept (at age 65).

### Software

The mixed models were fitted with the R package *lme4* (v. 1.1-21, Bates et al., 2015). Univariate (for influence measures) and bivariate latent growth curve models were fitted in Mplus (v. 8.4, Muthén and Muthén, 1998–2017)^1^. The R package *MplusAutomation* (v. 0.7-3, Hallquist and Wiley, 2018) was used to fit the bivariate models in a loop. Principle components were plotted with the R package *factorextra* (v. 1.0.5)^2^.

## Results

**FIGURE 2:**
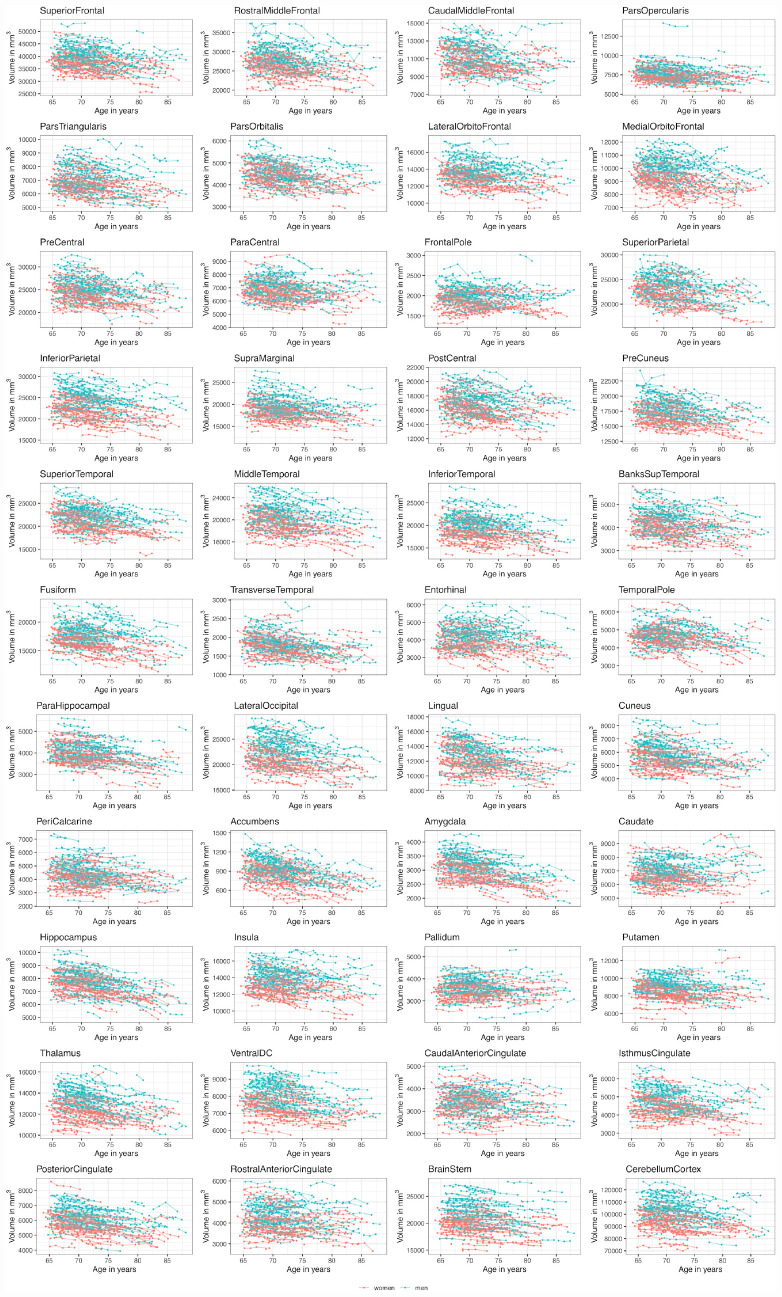
Volumetric brain structure trajectories (sum of left and right hemisphere) colored by sex.

The raw data trajectories of the different brain structures are shown Figure 2. Excluded subjects are shown in Supplementary Figure S1.

### Average Volumetric Declines

**FIGURE 3:**
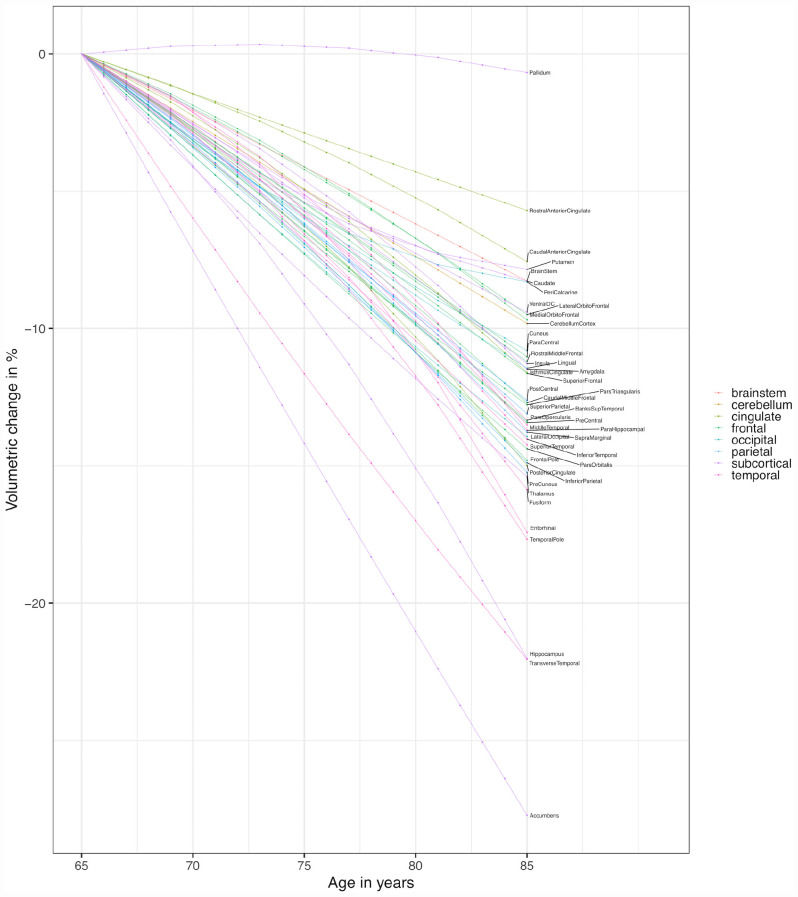
Expected structural slopes. The expected volumetric loss (of the average volumes at age 65) over the span of 20 years (starting at age 65 with an updated slope every 4 years until age 85) was extrapolated from the linear combination of the slope and the slope × entry-age interaction parameters.

The expected volumetric declines over the span of 20 years are shown in Figure 3. The expected slopes were extrapolated from the linear combination of the slope and the slope × entry-age interaction parameter, starting at age 65 with an updated slope every 4 years.

We observed some variability in the average volumetric decline of brain structures belonging to the frontal, temporal, and parietal lobe. However, generally, the expected declines from age 65 to age 85 of these regions seem to be in similar ranges (ranging from about 12 to 15% for the majority of structures belonging to the frontal, temporal or parietal lobe). Slightly smaller volumetric declines were estimated for the lateral orbitofrontal cortex and medial orbitofrontal cortex (10%), and the paracentral lobule, rostral middle frontal gyrus and superior frontal gyrus (∼11%). Slightly larger declines were estimated for the fusiform gyrus, entorhinal cortex, temporal pole, and transverse temporal gyrus (∼16 to 22%). Of the occipital regions, the peri-calcarine cortex showed the smallest decline (8%), followed by the cuneus and lingual gyrus (∼11%). The lateral occipital cortex showed a similar decline (14%) as structures of other lobes. Parameter estimates are shown in Table 1. Pairwise comparisons using bootstrap are shown in Supplementary Figure S2.

**TABLE 1:** Volumetric changes in % of brain structures average volumes (at age 65) with 95% confidence intervals.

Regions	Slope	Slope × Entry-Age	Loss 20 years	Random Slope SD	Random Slope *P* value	Sex *P* value
Caudal Middle Frontal	−0.556 (−0.398, −0.707)	−0.010 (0.013, −0.034)	−12.726 (−10.593, −14.891)	0.125 (0.008, 0.356)	0.563	0.074
Frontal Pole	−0.663 (−0.443, −0.871)	−0.010 (0.022, −0.044)	−14.831 (−11.681, −18.038)	0.458 (0.197, 0.656)	0.076	0.479
Lateral Orbitofrontal	−0.385 (−0.260, −0.508)	−0.011 (0.008, −0.031)	−9.546 (−7.719, −11.354)	0.108 (0.003, 0.288)	0.887	0.125
Medial Orbitofrontal	−0.363 (−0.178, −0.559)	−0.015 (0.012, −0.042)	−9.726 (−7.288, −12.093)	0.162 (0.007, 0.427)	0.937	0.371
Para-Central	−0.533 (−0.376, −0.686)	−0.002 (0.019, −0.025)	−11.065 (−8.933, −13.215)	0.173 (0.005, 0.360)	0.912	0.044
Pars Opercularis	−0.626 (−0.503, −0.751)	−0.005 (0.013, −0.024)	−13.394 (−11.692, −15.086)	0.154 (0.024, 0.303)	0.347	0.026
Pars Orbitalis	−0.737 (−0.597, −0.873)	0.002 (0.023, −0.020)	−14.437 (−12.487, −16.419)	0.001 (0.000, 0.291)	1.000	0.367
Pars Triangularis	−0.682 (−0.546, −0.812)	0.005 (0.026, −0.016)	−12.808 (−10.902, −14.795)	0.212 (0.085, 0.354)	0.003	0.168
Pre-Central	−0.631 (−0.486, −0.770)	−0.005 (0.016, −0.027)	−13.446 (−11.422, −15.498)	0.214 (0.013, 0.373)	0.359	0.051
Rostral Middle Frontal	−0.533 (−0.378, −0.690)	−0.003 (0.019, −0.027)	−11.261 (−9.235, −13.200)	0.043 (0.000, 0.109)	0.633	0.024
Superior Frontal	−0.548 (−0.401, −0.691)	−0.004 (0.017, −0.026)	−11.655 (−9.734, −13.585)	0.178 (0.012, 0.341)	0.342	0.010
Inferior Parietal	−0.677 (−0.563, −0.791)	−0.008 (0.008, −0.026)	−14.904 (−13.277, −16.545)	0.190 (0.011, 0.312)	0.487	0.113
Post-Central	−0.627 (−0.502, −0.749)	−0.000 (0.017, −0.018)	−12.633 (−10.944, −14.291)	0.194 (0.013, 0.338)	0.560	0.630
Pre-Cuneus	−0.641 (−0.533, −0.750)	−0.015 (0.002, −0.033)	−15.256 (−13.451, −17.065)	0.186 (0.012, 0.323)	0.548	0.015
Superior Parietal	−0.592 (−0.457, −0.725)	−0.008 (0.010, −0.027)	−13.117 (−11.419, −14.860)	0.072 (0.013, 0.299)	0.203	0.010
Supra Marginal	−0.577 (−0.463, −0.688)	−0.014 (0.002, −0.031)	−13.769 (−12.304, −15.320)	0.209 (0.023, 0.303)	0.143	0.015
Banks Sup Temporal	−0.505 (−0.390, −0.621)	−0.020 (−0.001, −0.040)	−13.354 (−11.381, −15.374)	0.223 (0.062, 0.341)	0.110	0.019
Entorhinal	−0.379 (−0.141, −0.629)	−0.062 (−0.027, −0.095)	−17.423 (−13.676, −21.288)	0.782 (0.613, 0.965)	3e-06	0.025
Fusiform	−0.564 (−0.457, −0.674)	−0.029 (−0.012, −0.046)	−15.856 (−14.099, −17.708)	0.210 (0.038, 0.328)	0.188	0.206
Inferior Temporal	−0.525 (−0.399, −0.647)	−0.022 (−0.003, −0.041)	−14.035 (−12.156, −15.990)	0.267 (0.099, 0.388)	0.105	0.062
Middle Temporal	−0.536 (−0.420, −0.652)	−0.017 (−0.001, −0.035)	−13.515 (−11.896, −15.192)	0.266 (0.168, 0.353)	0.019	0.069
Para-Hippocampal	−0.395 (−0.270, −0.522)	−0.036 (−0.015, −0.057)	−13.702 (−11.561, −15.930)	0.341 (0.224, 0.445)	0.004	0.033
Superior Temporal	−0.660 (−0.535, −0.786)	−0.006 (0.011, −0.024)	−14.250 (−12.696, −15.852)	0.224 (0.057, 0.341)	0.172	0.021
Temporal Pole	−0.545 (−0.357, −0.738)	−0.042 (−0.013, −0.073)	−17.623 (−14.605, −20.960)	0.598 (0.475, 0.721)	1e-08	0.300
Transverse Temporal	−1.206 (−1.032, −1.376)	0.013 (0.038, −0.013)	−22.089 (−19.577, −24.551)	0.211 (0.024, 0.422)	0.374	0.004
Cuneus	−0.624 (−0.492, −0.758)	0.011 (0.033, −0.012)	−10.844 (−8.562, −13.146)	0.213 (0.028, 0.399)	0.152	0.128
Lateral Occipital	−0.743 (−0.616, −0.869)	0.006 (0.026, −0.015)	−13.958 (−12.028, −15.979)	0.257 (0.103, 0.361)	0.068	0.433
Lingual	−0.618 (−0.502, −0.736)	0.006 (0.024, −0.012)	−11.457 (−9.581, −13.374)	0.340 (0.245, 0.446)	4e-04	0.101
Peri-calcarine	−0.674 (−0.446, −0.899)	0.032 (0.063, 0.003)	−8.330 (−5.440, −11.149)	0.490 (0.277, 0.668)	0.016	0.616
Caudal Anterior Cingulate	−0.282 (−0.154, −0.407)	−0.012 (0.005, −0.030)	−7.575 (−5.864, −9.296)	0.005 (0.004, 0.290)	0.992	0.010
Isthmus Cingulate	−0.437 (−0.309, −0.566)	−0.018 (0.005, −0.041)	−11.589 (−9.262, −13.941)	0.328 (0.216, 0.432)	0.002	0.026
Posterior Cingulate	−0.544 (−0.415, −0.672)	−0.026 (−0.004, −0.047)	−15.013 (−12.996, −17.032)	0.200 (0.018, 0.325)	0.457	0.005
Rostral Anterior Cingulate	−0.289 (−0.144, −0.439)	0.001 (0.022, −0.022)	−5.709 (−3.663, −7.796)	0.021 (0.001, 0.093)	0.900	0.013
Accumbens	−1.439 (−1.142, −1.750)	0.007 (0.047, −0.034)	−27.643 (−23.398, −32.031)	0.429 (0.105, 0.843)	0.063	0.956
Amygdala	−0.383 (−0.250, −0.517)	−0.024 (−0.003, −0.043)	−11.455 (−9.269, −13.621)	0.481 (0.392, 0.578)	6e-14	0.573
Caudate	−0.585 (−0.450, −0.718)	0.021 (0.044, −0.002)	−8.305 (−5.956, −10.606)	0.463 (0.385, 0.555)	3e-12	0.568
Hippocampus	−0.784 (−0.666, −0.904)	−0.040 (−0.018, −0.061)	−21.999 (−19.656, −24.339)	0.441 (0.366, 0.518)	1e-13	0.286
Insula	−0.499 (−0.374, −0.623)	−0.008 (0.011, −0.028)	−11.262 (−9.342, −13.373)	0.307 (0.198, 0.423)	0.003	0.016
Pallidum	0.069 (0.161, −0.024)	−0.013 (0.003, −0.029)	−0.703 (0.991, −2.446)	0.203 (0.065, 0.316)	0.230	0.986
Putamen	−0.639 (−0.529, −0.748)	0.031 (0.046, 0.016)	−7.850 (−6.318, −9.386)	0.326 (0.254, 0.405)	1e-05	0.697
Thalamus	−0.832 (−0.745, −0.920)	0.008 (0.021, −0.006)	−15.409 (−14.043, −16.777)	0.240 (0.170, 0.304)	3e-04	0.385
Ventral DC	−0.516 (−0.438, −0.593)	0.006 (0.018, −0.006)	−9.409 (−8.180, −10.632)	0.210 (0.146, 0.276)	0.001	0.016
Brain Stem	−0.411 (−0.362, −0.461)	−0.000 (0.007, −0.007)	−8.268 (−7.493, −9.028)	0.112 (0.042, 0.164)	0.075	0.607
Cerebellum Cortex	−0.494 (−0.418, −0.572)	0.000 (0.012, −0.011)	−9.818 (−8.654, −10.960)	0.147 (0.027, 0.219)	0.193	0.827

*Slope = expected volumetric annual changes at age 65. Slope × Entry-Age = interaction slope with entry-age. Loss 20 years = expected volumetric loss over the span of 20 years (starting at age 65 with an updated slope every 4 years until age 85) extrapolated from the linear combination of the slope and the slope × entry-age interaction parameters. Random Slope *P* value = *p*-value comparing a random slope model to a random intercept model with a likelihood ratio test. Sex *P* value = *p*-value obtained with a likelihood ratio test comparing a model including a separate slope and a separate slope × entry-age interaction parameter for men and women to a model without separate parameters.*

In most of the temporal, frontal, and parietal regions, an accelerating decline with advancing age was estimated (slope × entry-age parameter in Table 1). This effect was particularly large in the medial temporal lobe regions (fusiform gyrus, temporal pole, entorhinal cortex, and parahippocampal gyrus). In frontal and parietal regions, the acceleration was less pronounced. In these regions, zero was included in all of the 95% confidence intervals of the slope × entry-age parameter. In occipital regions, the decline seemed more steady (lateral occipital cortex, lingual gyrus) or even decelerating with advancing age (peri-calcarine cortex, cuneus). The slope × entry-age parameter estimates are plotted in Supplementary Figure S3.

Volumetric declines of the subcortical regions were heterogeneous. The pallidum showed almost no decline (1%), the putamen and the caudate showed small declines (∼8%), the amygdala and the insula showed a moderate decline (11%), the thalamus a slightly larger decline (15%), and the hippocampus (22%) and the nucleus accumbens (28%) showed a rather large decline in comparison to other regions. The nucleus accumbens showed the largest decline of all the observed brain structures, with a steady decline of about 1.4% per year. The declines of the hippocampus and the amygdala were clearly accelerating with advancing age, while the decline of the putamen and caudate was decelerating with advancing age.

### Sex Differences in Trajectories

Given that the estimated total ICV was already included (Table 1, the *x*-axis of Supplementary Figure S4), allowing for different slopes and different slope × entry-age interactions for men and women did not substantially improve the model fit for most of the brain structures. We would like to emphasize that using ICV as covariate usually eliminates most of the sex influences on brain volume measures (Jäncke et al., 2015, 2019). As expected, the estimated influence of sex were small in comparison with the general slopes at age 70 (slope parameter of the *y*-axis of Supplementary Figure S4 and Supplementary Table S2). In sum, the data provide little (or even no) evidence that one of the sexes declines faster than the other in the examined age range considering the sample size, the number of comparisons, the observational nature of the study, and the freedom in modeling such trajectories.

### Random Slope Variances

**FIGURE 4:**
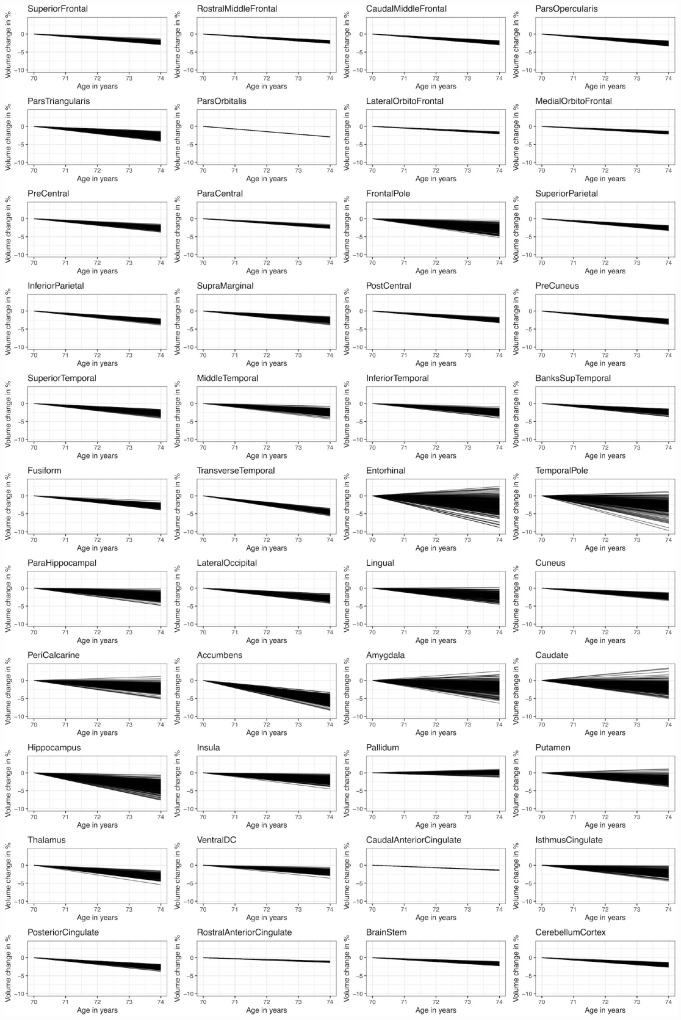
Estimated person-specific slopes in comparison to the average slope at an age of 70 years. *Y*-axis represents the% of average volume (at age 65) change. The random slopes were added to the average slope at an age of 70 years.

The estimated random slope variance parameters from the random slope models (with the following covariates for the slope parameter: entry-age, total-ICV, sex and sex × entry-age) are shown in Table 1 and in Supplementary Figure S5. Reliable (*p* < 0.05, Table 1) random slope variability was observed in subcortical regions (amygdala, caudate, hippocampus, putamen, thalamus, insula), in medial temporal lobe structures and temporal pole, and in lingual gyrus and peri-calcarine cortex with random slope standard deviations typically ranging between 0.2 and 0.5%. The largest variability was observed in the entorhinal cortex (0.8%), followed by the temporal pole (0.6%), followed by the amygdala, caudate, and hippocampus (∼0.5%). Model fit (evaluated with the likelihood ratio test) clearly improved in these regions when including the random slope parameter. Large variability was further observed in the peri-calcarine cortex, frontal pole, and nucleus accumbens (∼0.5%). However, the model fit did not improve clearly for these regions due to large uncertainty in the estimated random slopes. In most regions of the frontal lobe, the parietal lobe, and the cingulum the random slope standard deviation estimate ranged from 0 to 0.2%. Allowing for random slopes did not substantially improve the model fit in these regions. To illustrate the random slope variation in the expected annual changes, the estimated random slopes are plotted alongside the average slopes at an age of 70 years in Figure 4. In frontal and parietal regions, some subjects were expected to have little decline (∼1%) while others seem to be clearly declining (∼4%) over the span of 4 years. In regions with reliable random slope differences (e.g., hippocampus), some subjects showed almost no decline while others were declining about twice as fast as the average slope.

### Associations Between Random Slopes

**FIGURE 5:**
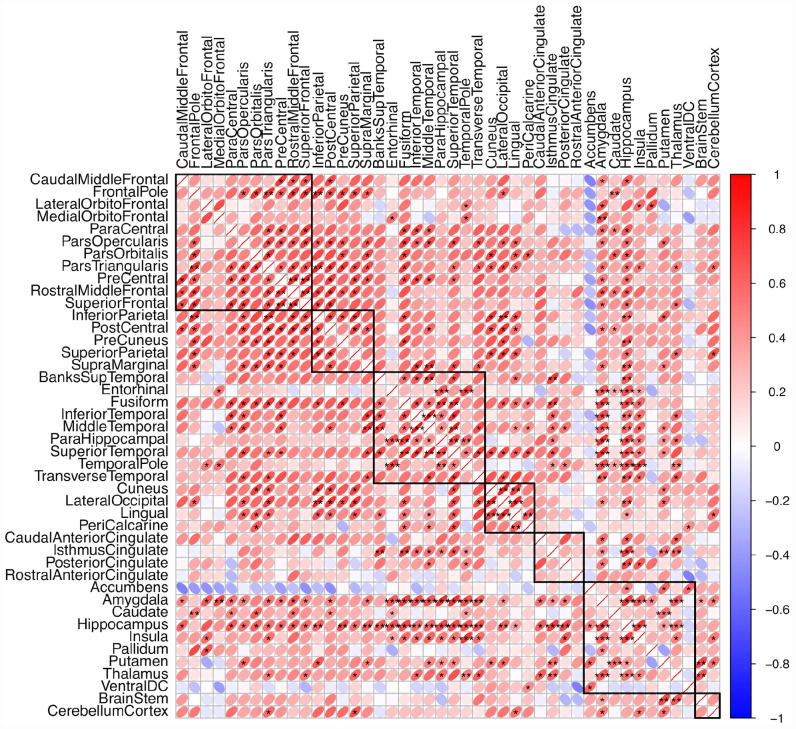
Medians of posterior distributions of correlations between the random slopes of different brain structures. The variance of the posteriors are quite large for many brain structures (as can indirectly be seen when the median is rather far away from zero, but a substantial mass of the posterior distribution is on the other side of zero; * indicates a posterior mass on the other side of zero < 0.05, ** corresponds to < 0.005, *** corresponds to < 0.0005).

Associations between the random slopes were estimated in bivariate models using Bayesian estimation with the default priors as provided by *Mplus*. Figure 5 shows the medians of the posterior distributions of the correlation parameter between random slopes from the bivariate models. The exact numbers are shown in Supplementary Figure S6.

In general, there are strong associations between the random slopes within and between the frontal, temporal, parietal and to a lesser extent occipital lobe with estimated correlations typically ranging between 0.5 and 0.9. However, many estimates are not reliable because associations between the slopes estimated for brain structures with little estimated random slope variance (compared to the within-subject error term) resulted in posterior distributions with a large variance under the assumed model. As can be seen in Figure 5, this was the case for a lot of structures.

There are a few exceptions to this homogenous correlation pattern. While the entorhinal cortex, parahippocampal gyrus and temporal pole showed strong and reliable associations with each other, with the hippocampus, and the amygdala, and strong but less reliable associations with other regions (the insula, lateral orbitofrontal and medial orbitofrontal cortex), they were only weakly correlated with most of the other brain structures. Regions from the lateral medial orbital and lateral orbitofrontal cortex showed rather strong but unreliable correlations with each other and further regions (with the temporal pole, with the entorhinal cortex, with the amygdala and with the hippocampus), but weak correlations with most other brain structures. The peri-calcarine cortex showed moderate correlations with other occipital structures and rather weak correlations to other lobe structures.

The correlation pattern involving subcortical structures was in general more diverse. Amygdala, hippocampus, and to a slightly lesser extent insula and thalamus, were strongly and reliably correlated with each other and with temporal lobe structures, but also quite strongly with most other brain structures. The pallidum showed weak correlations with other structures. Putamen and caudate showed strong and reliable correlations with each other but weak or unreliable correlations with other structures. Nucleus accumbens stands out of the pattern because it was negatively correlated with most brain structures. Positive correlations for the nucleus accumbens were limited to the putamen, caudate and ventral DC (diencephalon). However, the estimates involving the nucleus accumbens were unreliable.

To obtain a simple estimate of the multivariate correlation structure on which it was possible to do PCA, we used the mean of the means from the plausible values distributions of the bivariate models. The estimated multivariate correlation matrix of these random slope estimates is shown in Supplementary Figure S7. In general, the correlations were slightly smaller than the ones estimated in the bivariate models but the pattern looked similar. This suggests that the explained variance in PCA may be underestimated, but that the direction of the principle components was reasonably approximated.

**FIGURE 6:**
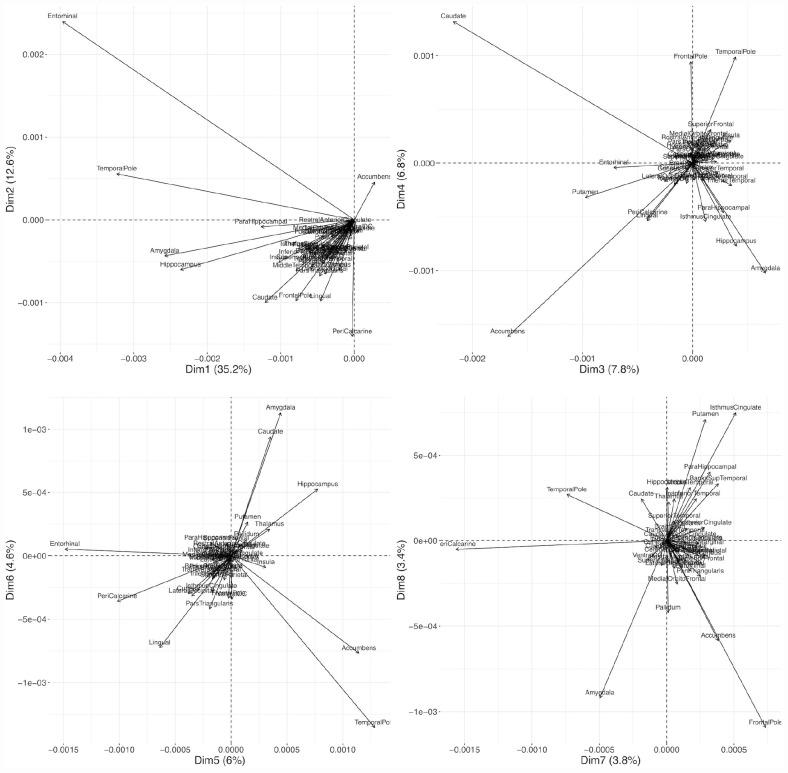
Biplots of the first eight principal components. PCA was applied to the covariance matrix of the expected random slopes.

Because the data were on similar scales already through the scaling by the intercept in each region, we applied the PCA on the unscaled person-specific slope estimates. The first principle component explained about 35% of the between-person variance of the random slopes and can be seen as a weighted average of most brain regions. It loads strongly on the entorhinal cortex, temporal pole, hippocampus, and amygdala because these regions had large random slope variances. The second principle component (explaining 12% of the slope variances), separates the entorhinal cortex, the temporal pole and the nucleus accumbens from most other structures, indicating that after the general decline has been accounted for, there is still a lot of variability in our sample in these regions (Figure 6 and Supplementary Table S3). The third principle component loads strongly on caudate, and nucleus accumbens, but also on putamen, entorhinal cortex, peri-calcarine cortex, and lingual gyrus, and separates these regions from most other regions (especially amygdala). The fourth component separates the nucleus accumbens, amygdala, and hippocampus from caudate, frontal pole, and temporal pole. The first 10 principal components explained already 85% of the 44 (brain structure) random slope variances.

## Discussion

Our study extends the current knowledge about brain structure trajectories by providing information about healthy aging from a longitudinal study with four repeated measurements and large sample size. In addition to describing the average brain volume trajectories, we put particular emphasis on the variation of the person-specific trajectories and the associations between the person-specific trajectories.

### Brain Regions With Largest Decline

Previous research presents a heterogeneous picture about which brain structures are most affected by the volumetric decline in aging. Frontal and temporal lobes were reported to show the largest decline in a large sample size study by Vinke et al. (2018). Other studies report some regional variability in the average volumetric decline but not a clear preference regarding largest volumetric losses for one lobe or lobular region, whereby regions of the occipital lobe were often found to show the smallest decline of the lobular regions (Driscoll et al., 2009; Fjell et al., 2009; Raz et al., 2010, 2005; Pfefferbaum et al., 2013; Storsve et al., 2014). This is in accordance with our data that showed some variability in the average decline in regions of the frontal, temporal and parietal lobes, but in general, these brain regions showed similar volumetric reductions. Entorhinal cortex, temporal pole and transverse temporal gyrus showed slightly larger declines, while half of the regions belonging to the frontal lobe (lateral and medial orbitofrontal cortex, paracentral lobule, rostral middle frontal gyrus and superior frontal gyrus) and regions belonging to the occipital lobe (peri-calcarine gyrus, cuneus, lingual gyrus) showed slightly smaller volumetric reductions than other brain structures.

Subcortical brain structures seem to differ most with respect to their estimated decline trajectories over 20 years. The hippocampus has often been reported to be one of the brain regions with the largest decline and with an accelerating decline with increasing age (Raz et al., 2010, 2005; Fjell et al., 2013, 2009; Narvacan et al., 2017; Vinke et al., 2018). In our data, the hippocampus showed one of the largest volumetric reductions of all the observed brain regions, and the decline was clearly accelerating with advancing age. Further, the amygdala has often been reported to show an accelerated decline with increasing age (Fjell et al., 2013, 2009; Narvacan et al., 2017), which is also consistent with our data.

In our analyses, the nucleus accumbens showed the steepest decline of all the observed brain structures with no acceleration with age. While this is in line with the studies by Coupé et al. (2017) and Fjell et al. (2013), at least one other study proposed an alternative trajectory shape (U-shaped curve; Vinke et al., 2018). The putamen and the caudate have been reported to show slight increases in very old age (Fjell et al., 2013; Coupé et al., 2017). In our data, the slopes of these two regions (especially of the putamen) were decelerating with increasing age. The pallidum showed almost no decline in our study, which was similarly reported in Narvacan et al. (2017), but which is in contrast to the large study by Vinke et al. (2018).

Of note, studies differ in their ways of data scaling. We scaled our data by the average volume at age 65. The percent of volumetric decline depends on data scaling and on the studied age range. This complicates direct comparison to other studies. Another important aspect is that we did not observe subjects over the span of 20 years but estimated the expected decline over 20 years based on the slope and the slope × entry-age parameters. The estimates should be less noisy than in cross-sectional studies, but there is still more noise in these estimates than it would be the case if subjects had been observed over the span of 20 years.

### Sex Differences

Previous studies yielded mixed results regarding the question of whether men differ from women in their aging trajectories. Vinke et al. (2018) report statistically significant age × sex interactions in all studied brain regions. Coupé et al. (2017) found sex differences in individuals beyond age 70 when using brain volumes normalized by the ICV. The few studies that separated the between-subject from the within-subject effects generally found no or only little evidence for sex differences in the studied regions (Raz et al., 2010, 2005; Pfefferbaum et al., 2013; Narvacan et al., 2017). Our analyses, in which ICV was included as an additional covariate, do not provide convincing evidence for sex differences in the slopes in almost all studied brain regions. Based on our data and on previous studies, it seems that the sex differences in the expected trajectories are rather small. The estimates, however, may depend on the assumed model e.g., whether we assume a linear and additive model including the covariates ICV and sex or whether the brain regions submitted to the models are normalized by ICV (e.g., Narvacan et al., 2017 used both measures). Nevertheless, there may be other factors, such as different lifestyle choices, which have a bigger influence on the trajectories (Raz et al., 2010, 2005). We included the sex covariate in the models that were used to estimate the correlation parameters based on theoretical considerations, even though they had little or almost no impact on the estimated correlations.

### Random Slope Variances

Quantifying random slope variances of the different brain structures has not been of major interest in most studies to date, except for two studies by Raz et al. (2010, 2005). Reliable random slope variances have been reported in almost all of their studied regions (except the primary occipital and the orbital-frontal cortex). However, the random slope variances in the studies by Raz et al. might have been overestimated given that a clean separation of the within-person error from the random slope term is difficult in case of only two repeated measures (Grimm et al., 2017). Our data, with four measurement occasions, allow to more precisely disentangle the within-person error from the random slope and, thus, lead to more accurate estimates of the random slope variances.

Evaluating our data, reliable random slope variability was observed in subcortical regions, in medial temporal structures and temporal pole, and in lingual gyrus and peri-calcarine cortex. Because the within-subject error is quite large compared to the annual volumetric changes and the person-specific variations thereof, the person-specific slopes and the random slope variance term were estimated with large uncertainty in a lot of regions. Therefore, the conclusion that some regions have little random slope variability and other regions have large variability needs to be taken with caution. Nevertheless, regions belonging to the medial temporal cortex (hippocampus, entorhinal cortex, parahippocampal gyrus), temporal pole, amygdala, and caudate showed reliable random slope variability, and in the population, the random slope variance may be rather large. In these regions, some subjects showed almost no decline while other subjects were declining twice as fast as the average person. However, such a variation of the decline rate may be present in most other brain structures as well, including those with unreliably estimated random slope variances.

### Associations Between Random Slopes

One of the main objectives of this study was to investigate the associations of the slopes of different brain structures – an important aspect that was analyzed by Raz et al. (2005), but that has not been of much interest since then. In their study, estimates were mostly positive but also rather small in magnitude. Reliable associations were only found for 13 of 55 studied slope-to-slope associations. However, we think that these correlations were probably underestimated because the correlation estimates are influenced by the noise of the within-subject error term. Our data suggest that the slope-to-slope correlation pattern is, in general, quite homogenous, especially within but also between most regions of the frontal, temporal and parietal lobe and, to a lesser extent, of the occipital lobe, while the correlation pattern of subcortical regions was more diverse. Besides subcortical structures, there are a few lobular structures that divert this homogenous correlation pattern. Of special interest are the entorhinal cortex and the temporal pole, which were strongly and reliably associated with each other, with the amygdala, with the hippocampus, and with the insula but showed only weak correlations with other lobular structures. However, correlations involving structures with unreliable random slope variances (the majority of regions of frontal and parietal lobe structures) are often broad and often substantially overlap zero, even when the median indicates a large correlation. In the maximum likelihood estimation framework, these models would often lead to non-positive definite covariance matrix warnings, which may then be resolved by setting the random slope variance to zero.

### Modeling Considerations

There is a lot of between-subject variability in the size of brain volumes. Separating the between-subject from the within-subject effect is important (Grimm et al., 2016). It can be achieved by separating the age of the participants into the age at the study entry and the difference from the age at study entry to the age at subsequent measurements (e.g., as done in Pfefferbaum et al., 2013). Such an approach was not always applied in previous longitudinal studies, and it seems to be worth considering it in further analyses. Aside from this, the estimation of non-linear trajectories is only reasonable with four or more time points, which highlights the need for longitudinal studies with more time points. In our analyses, we refrained from including a quadratic slope because it would have made the interpretation of the slope parameters more difficult (e.g., a linear and a quadratic random slope may cancel each other out in a person) and because the first and the last measurement were only 4 years apart. Also, four time points are still at the lower limit to appropriately capture person-specific trajectories. We are aware that our rather simple linear models did not capture the true trajectories in great detail, but we expect them to give a reasonable approximation. Model fit indices further indicated no grossly miss-specified models. Further, simpler models are less prone to overfitting. However, more time points and a longer observational time span would clearly be beneficial in many ways.

### Measurement Error Term and Image Quality

A large amount of the uncertainty in the random slope variances and covariances is due to the large within-subject error term. This within-subject error consists of true deviations from the assumed linear shape and of the measurement errors related to the structural MRI scanning procedure (e.g., artifacts) and data processing. The measurement errors are probably responsible for the main part of the within-subject variance. Therefore, the reduction of the measurement errors would substantially improve estimation. Motion artifacts and deterioration of scan quality have been proposed to confound estimates attributed to age effects in cross-sectional studies (Alexander-Bloch et al., 2016; Ducharme et al., 2016; Rosen et al., 2018; Klapwijk et al., 2019). Systematic changes in image quality during repeated measures may also confound parameter estimates in longitudinal studies. In addition, inclusion of such motion and scan quality regressors in the models may be able to reduce the measurement error variance. If no such movement data were recorded during T1 acquisition then an approximate movement regressor may be estimated from functional MRI data acquired during the same scanning session (Alexander-Bloch et al., 2016). However, we think that the inclusion of such approximate motion and scan quality regressors are of little help to improve the estimation of the slope parameters. First, these approximate regressors are relatively imprecise. Second, in a longitudinal study, it may be reasonable to assume that individuals show similar motion and rather a stable image quality during repeated measures. Finally, inclusion of time varying regressors that show systematic change may distort the estimates, making interpretation more difficult. An option would be to model these regressors as growth processes and to regress their random intercepts and slopes on the random intercepts and slopes of the brain structures. We tested this approach on some brain structures by using the Euler number as a proxy for image quality (Rosen et al., 2018). Modeling the Euler number as a growth process, it showed decreases over the 4 years and a slope × entry-age interaction (larger decreases with advancing entry-age) with unreliable random slopes. The inclusion of the Euler number as a growth process in the models lead to slightly higher uncertainty but similar covariance estimates as in the reported models. The slope × entry-age interaction estimates were slightly lower in magnitude as in the reported models. Worthy of note, this approach increases model complexity by a large amount and may lead to convergence issues. We conclude from these additional analyses, that the estimates of this study should be taken with caution, and that the uncertainty of these estimates might be even higher than our fitted models propose.

### Definition of Healthy Aging

The population of interest in our study was healthy elderly people, where the definition of “healthy” was based on the inclusion criteria. It seems clear that there is still a lot of variability in the term healthy aging. Lifestyle factors (like physical activity) that may influence the volumetric trajectories have not been considered in this analysis. For example, there is some evidence that physical activity positively influences changes in hippocampal volume (Erickson et al., 2011; Varma et al., 2015). Pre-symptomatic Alzheimer pathology may influence trajectories negatively (Fjell et al., 2013). Estimates of brain structure trajectories − especially about the variability and the associations between random slopes − may critically depend on the definition of healthy aging.

## Conclusion

Expected volumetric brain region changes, as well as variations and associations of random slopes, have been described in this paper. The focus on random slope variations and associations thereof provided insights into the heterogeneity of the aging process. We think this is valuable information that should not be missed when analyzing such data. Our data present a picture of a rather homogenous aging process of volumetric brain region changes. Subcortical regions, medial temporal structures, and the temporal pole showed reliable random slope variances and a more diverse correlation pattern. Data from further studies with more repeated measures and a longer observational time span would be beneficial.

## Data Availability Statement

The R analysis code and the associated data tables will be made available by the authors. The raw MR image data underlying this article are not publicly available and can only be accessed via collaborations with the URPP Dynamics of Healthy Aging.

## Ethics Statement

The studies involving human participants were reviewed and approved by Kantonale Ethikkommission Zürich (EK 2010-0267/3). The patients/participants provided their written informed consent to participate in this study.

## Author Contributions

SM, FL, and LJ contributed to the design, set-up, maintenance, and support of the Longitudinal Healthy Aging Brain (LHAB) database. SS performed the statistical analysis and wrote the first draft of the manuscript. All authors contributed to manuscript revision and approved the submitted version.

## Conflict of Interest

The authors declare that the research was conducted in the absence of any commercial or financial relationships that could be construed as a potential conflict of interest.

## Footnotes

1 https://www.statmodel.com

2 https://CRAN.R-project.org/package=factoextra
