# Supplementary material for: Decline Variability of Cortical and Subcortical Regions in Aging: A Longitudinal Study
Source: Front Hum Neurosci. 2020 Sep 4;14:363. doi: 10.3389/fnhum.2020.00363 (PMC7500514; doi:10.3389/fnhum.2020.00363)
Supplement: Supplementary file 1 [file Data_Sheet_1.PDF]

Table S1: Missing data patterns. X = not missing. E.g. 22 subjects were measured on all time points. 138 were measured on all timepoints except the 3 year-follow up. The frequency sums up to N = 231.

Baseline	X	X	X	X	X	X	X	X	X
1-year follow-up	X	X		X	X		X	X	
2-year follow-up	X	X			X	X	X		X
3-year follow-up	X						X		
4-year follow-up	X	X				X		X	
Frequency	22	138	18	15	28	4	2	2	2

Table S2: Volumetric changes in % of brain structures average volumes (at age 65) seprated for men and women. SlopeWomen represents the average slope for women at age 65. SlopeMen represents the average slopes for men at age 65. SlopeXAgeEntryWomen represents the slope x age-entry interaction parameter for women. SlopeXAgeEntryMen represents the slope x age-entry interaction parameter for men. The p-value was obtained with a likelihood ratio test comparing a model including a separate slope and a separate slope x age-entry interaction parameter for men and women to a model without separate parameters.

Regions	SlopeWomen	SlopeMen	SlopeXEntryAgeWomen	SlopeXEntryAgeMen	Pval
CaudalMiddleFrontal	-0.763	-0.375	0.012	-0.028	0.030
FrontalPole	-0.680	-0.670	-0.018	0.002	0.623
LateralOrbitoFrontal	-0.446	-0.351	-0.014	-0.006	0.275
MedialOrbitoFrontal	-0.416	-0.333	-0.018	-0.011	0.644
ParaCentral	-0.653	-0.456	-0.002	0.003	0.128
ParsOpercularis	-0.750	-0.538	-0.000	-0.006	0.080
ParsOrbitalis	-0.797	-0.693	0.005	0.002	0.659
ParsTriangularis	-0.825	-0.553	0.020	-0.008	0.111
PreCentral	-0.777	-0.512	0.003	-0.010	0.115
RostralMiddleFrontal	-0.700	-0.408	0.005	-0.007	0.066
SuperiorFrontal	-0.697	-0.435	-0.002	-0.003	0.036
InferiorParietal	-0.790	-0.582	0.000	-0.015	0.178
PostCentral	-0.628	-0.638	-0.005	0.005	0.737
PreCuneus	-0.783	-0.530	-0.009	-0.018	0.044
SuperiorParietal	-0.774	-0.458	0.002	-0.012	0.027
SupraMarginal	-0.727	-0.455	-0.004	-0.020	0.029
BanksSupTemporal	-0.622	-0.426	-0.019	-0.017	0.065
Entorhinal	-0.592	-0.255	-0.067	-0.045	0.067
Fusiform	-0.662	-0.482	-0.021	-0.035	0.313
InferiorTemporal	-0.623	-0.457	-0.021	-0.019	0.174
MiddleTemporal	-0.628	-0.470	-0.016	-0.017	0.190
ParaHippocampal	-0.450	-0.383	-0.048	-0.020	0.033
SuperiorTemporal	-0.727	-0.632	-0.014	0.006	0.030
TemporalPole	-0.587	-0.530	-0.050	-0.032	0.462
TransverseTemporal	-1.342	-1.157	0.002	0.036	0.005
Cuneus	-0.729	-0.550	0.015	0.010	0.307
LateralOccipital	-0.789	-0.711	0.007	0.006	0.734
Lingual	-0.707	-0.559	0.007	0.008	0.260
PeriCalcarine	-0.658	-0.711	0.020	0.046	0.600
CaudalAnteriorCingulate	-0.419	-0.193	-0.011	-0.007	0.035
IsthmusCingulate	-0.670	-0.236	0.005	-0.037	0.006
PosteriorCingulate	-0.740	-0.393	-0.015	-0.032	0.011
RostralAnteriorCingulate	-0.432	-0.196	0.001	0.006	0.046
Accumbens	-1.556	-1.341	0.028	-0.011	0.666
Amygdala	-0.337	-0.439	-0.039	-0.008	0.240
Caudate	-0.419	-0.740	-0.006	0.046	0.031
Hippocampus	-0.804	-0.786	-0.047	-0.030	0.365
Insula	-0.572	-0.460	-0.017	0.005	0.025
Pallidum	0.099	0.036	-0.019	-0.007	0.710
Putamen	-0.618	-0.656	0.030	0.031	0.925
Thalamus	-0.753	-0.907	-0.002	0.017	0.214
VentralDC	-0.424	-0.586	0.003	0.006	0.053
BrainStem	-0.388	-0.431	-0.003	0.002	0.726
CerebellumCortex	-0.500	-0.491	0.000	0.001	0.973

Table S3: PCA Loadings of the first twelve principal components.

	PC1	PC2	PC3	PC4	PC5	PC6	PC7	PC8	PC9	PC10	PC11	PC12
CaudalMiddleFrontal	-0.04	-0.10	0.04	0.01	-0.02	0.01	0.03	-0.03	0.05	-0.04	-0.01	-0.00
FrontalPole	-0.11	-0.23	-0.01	0.30	-0.01	-0.13	0.32	-0.50	0.12	-0.02	-0.35	-0.35
LateralOrbitoFrontal	-0.04	-0.05	0.02	0.03	-0.01	-0.03	0.03	-0.07	0.02	0.00	0.09	-0.09
MedialOrbitoFrontal	-0.06	-0.04	0.02	0.07	-0.06	-0.01	0.03	-0.12	-0.03	0.00	0.12	-0.14
ParaCentral	-0.05	-0.11	0.03	0.04	-0.03	0.00	0.04	-0.03	0.03	-0.05	0.05	0.12
ParsOpercularis	-0.05	-0.09	0.02	0.01	-0.06	-0.05	0.10	-0.01	-0.00	-0.02	0.04	0.23
ParsOrbitalis	-0.04	-0.06	-0.00	0.03	-0.02	-0.05	0.04	-0.03	0.01	-0.01	0.02	0.04
ParsTriangularis	-0.07	-0.16	-0.00	0.05	-0.07	-0.16	0.11	-0.10	-0.12	0.16	0.12	0.53
PreCentral	-0.07	-0.12	0.06	0.05	-0.03	0.01	0.10	-0.04	0.05	-0.20	0.02	0.07
RostralMiddleFrontal	-0.03	-0.10	0.01	0.04	-0.01	-0.02	0.04	-0.06	0.02	0.02	0.01	0.01
SuperiorFrontal	-0.06	-0.09	0.05	0.10	-0.02	0.04	0.11	-0.06	0.05	-0.12	-0.01	-0.02
InferiorParietal	-0.05	-0.11	-0.01	0.01	-0.06	-0.07	0.12	-0.04	0.01	0.03	-0.03	0.00
PostCentral	-0.05	-0.14	0.02	0.05	-0.04	-0.01	0.05	-0.07	0.04	-0.06	-0.01	0.09
PreCuneus	-0.05	-0.10	-0.00	-0.00	-0.03	-0.06	0.12	-0.05	-0.01	0.05	0.06	-0.06
SuperiorParietal	-0.02	-0.09	-0.02	0.00	-0.02	-0.08	-0.02	-0.07	0.03	0.08	-0.02	0.07
SupraMarginal	-0.06	-0.12	0.06	0.01	-0.04	-0.01	0.11	-0.02	0.06	-0.14	-0.01	0.08
BanksSupTemporal	-0.06	-0.10	0.03	-0.05	-0.05	-0.05	0.16	0.15	0.00	-0.12	0.08	-0.03
Entorhinal	-0.56	0.57	-0.22	-0.01	-0.51	0.02	0.08	-0.08	0.13	-0.04	-0.06	0.05
Fusiform	-0.09	-0.09	0.04	-0.05	-0.05	-0.07	0.07	0.03	-0.09	-0.04	0.09	-0.00
InferiorTemporal	-0.10	-0.11	0.11	-0.07	-0.06	0.00	0.10	0.11	-0.04	-0.23	0.02	0.10
MiddleTemporal	-0.11	-0.15	0.09	-0.06	-0.06	-0.02	0.08	0.14	-0.02	-0.27	-0.04	0.17
ParaHippocampal	-0.18	-0.02	0.08	-0.15	-0.06	0.04	0.14	0.18	-0.18	-0.23	0.22	-0.19
SuperiorTemporal	-0.10	-0.12	0.07	-0.03	-0.05	-0.02	0.02	0.05	-0.02	-0.18	0.02	0.06
TemporalPole	-0.46	0.13	0.12	0.32	0.44	-0.53	-0.32	0.12	-0.16	0.02	-0.04	-0.02
TransverseTemporal	-0.06	-0.13	-0.02	-0.06	-0.06	-0.06	0.03	0.01	-0.02	0.01	0.09	0.18
Cuneus	-0.03	-0.14	-0.06	-0.03	-0.13	-0.12	-0.04	-0.02	-0.06	0.18	0.04	0.00
LateralOccipital	-0.06	-0.15	-0.08	-0.05	-0.12	-0.12	0.07	-0.04	-0.02	0.24	-0.00	0.02
Lingual	-0.07	-0.23	-0.12	-0.17	-0.22	-0.28	-0.01	-0.01	-0.15	0.38	0.07	0.02
PeriCalcarine	-0.00	-0.33	-0.12	-0.16	-0.35	-0.14	-0.67	-0.02	0.20	-0.27	-0.10	-0.20
CaudalAnteriorCingulate	-0.03	-0.04	0.03	0.01	0.01	0.01	0.04	0.00	0.01	-0.04	0.01	-0.02
IsthmusCingulate	-0.09	-0.08	0.04	-0.18	-0.06	-0.11	0.22	0.34	-0.12	0.22	0.20	-0.52
PosteriorCingulate	-0.06	-0.05	0.07	0.00	0.01	-0.01	0.12	0.03	0.02	-0.11	0.10	-0.06
RostralAnteriorCingulate	-0.04	-0.02	0.01	0.06	-0.01	0.02	0.06	-0.01	0.00	-0.03	0.01	-0.08
Accumbens	0.04	0.11	-0.51	-0.52	0.39	-0.30	0.17	-0.27	0.07	-0.27	0.11	-0.01
Amygdala	-0.37	-0.10	0.20	-0.33	0.15	0.44	-0.21	-0.42	-0.46	0.08	0.02	-0.02
Caudate	-0.17	-0.24	-0.66	0.42	0.12	0.37	-0.08	0.11	-0.04	0.01	0.33	-0.02
Hippocampus	-0.33	-0.14	0.12	-0.25	0.27	0.20	0.00	0.14	0.70	0.33	-0.03	0.06
Insula	-0.15	-0.12	0.10	0.07	0.11	-0.04	0.02	0.11	0.08	-0.26	0.09	0.02
Pallidum	-0.01	-0.05	0.05	0.06	0.05	0.05	0.00	-0.19	0.08	-0.06	0.13	-0.11
Putamen	-0.08	-0.13	-0.29	-0.10	0.05	0.10	0.12	0.33	-0.25	-0.00	-0.69	0.05
Thalamus	-0.11	-0.08	0.00	-0.05	0.12	0.08	0.01	0.10	-0.01	-0.07	-0.20	-0.02
VentralDC	0.01	-0.03	-0.05	-0.06	0.00	-0.13	-0.06	-0.05	0.06	-0.01	0.06	0.10
BrainStem	-0.03	-0.04	-0.02	-0.02	0.03	0.02	0.01	0.02	0.01	-0.02	-0.08	0.02
CerebellumCortex	-0.04	-0.05	-0.02	-0.03	0.00	-0.02	0.01	-0.04	-0.00	0.08	-0.11	-0.01
Cumulative Variance explained	0.35	0.48	0.56	0.62	0.68	0.73	0.77	0.80	0.83	0.85	0.87	0.88

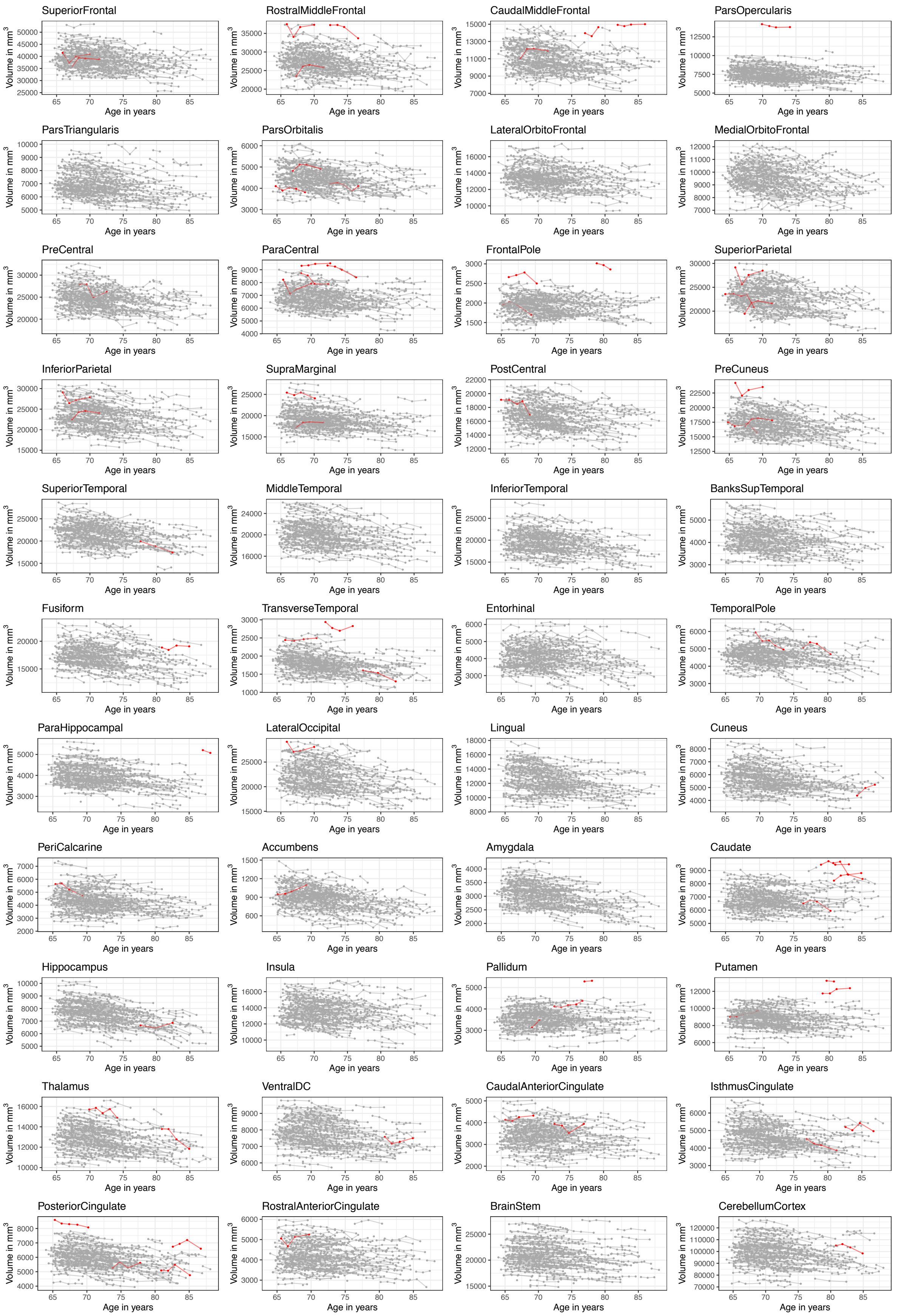

Figure S1: Brain trajectories of excluded subjects colored in red. Subjects were excluded because they had strong influence on model parameters based on Cooks distance and the loglikelihood contribution.

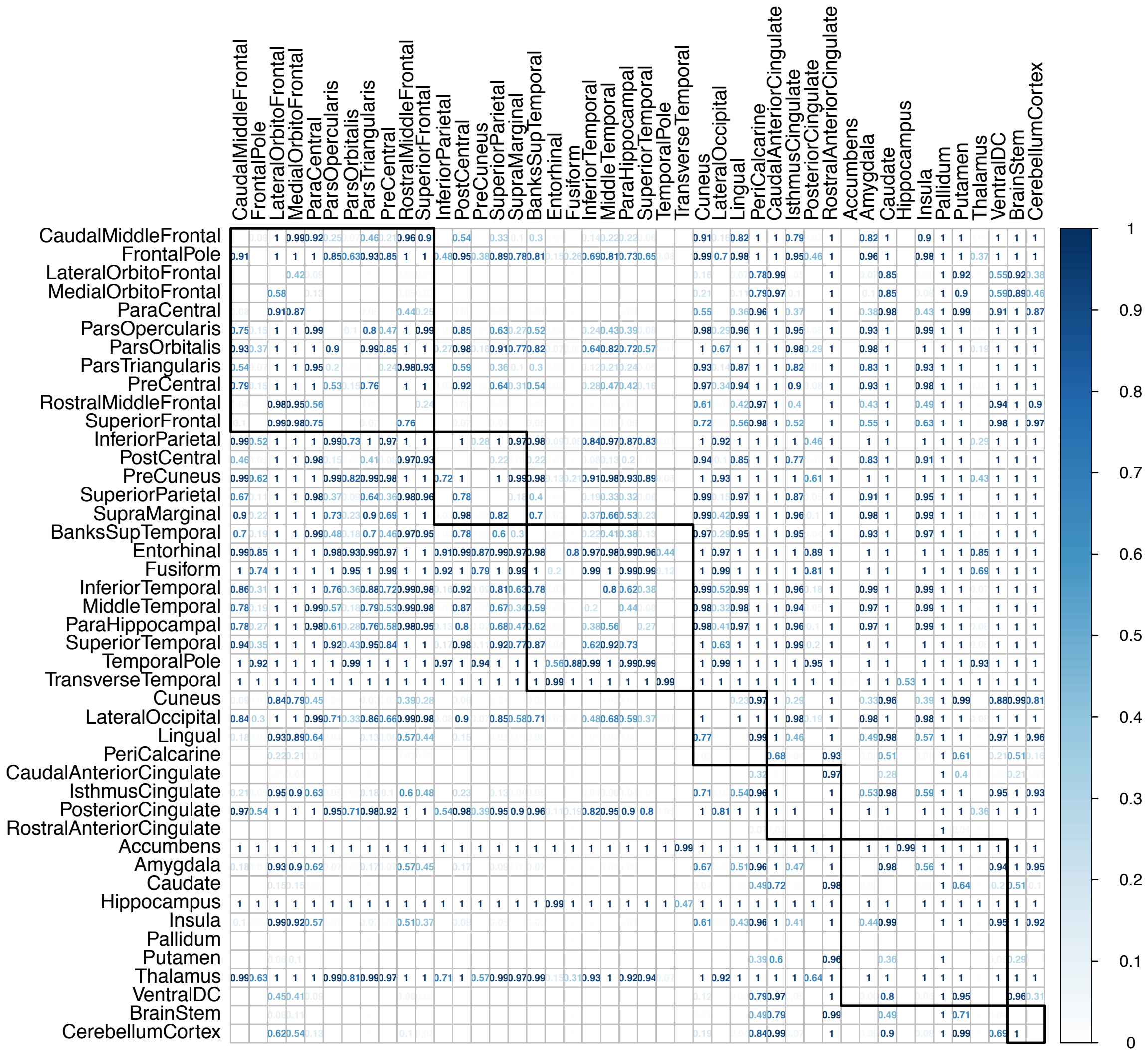

Figure S2: Bootstrap (N=10000) of pairwise average volumetric declines over 20 years. A bootstrapped mass of Region (Row) > Region (Column) was obtained by calculating the percent of bootstrap samples where the decline in region (Row) was estimated to be larger than the decline in region (Column). E.g. hippocampus (row) vs. frontal pole (Column): In 100% of the bootstrap samples, the estimated average decline of the hippocampus were larger than the estimated average decline from the frontal pole.

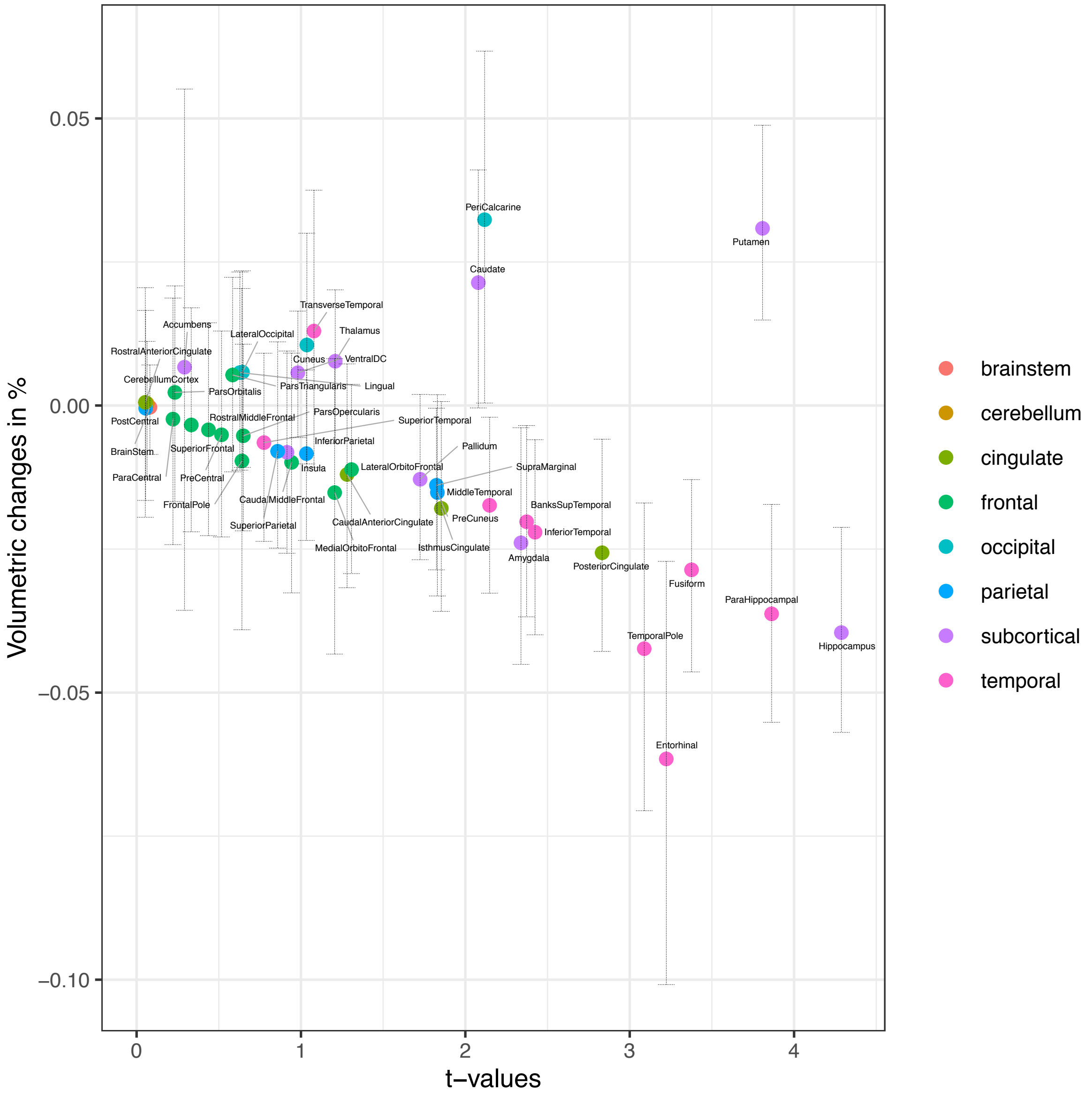

Figure S3: Slope x Entry-Age interaction parameter. X-axis represents the t-value comparing the slope x entry-age interaction parameter estimate to zero.

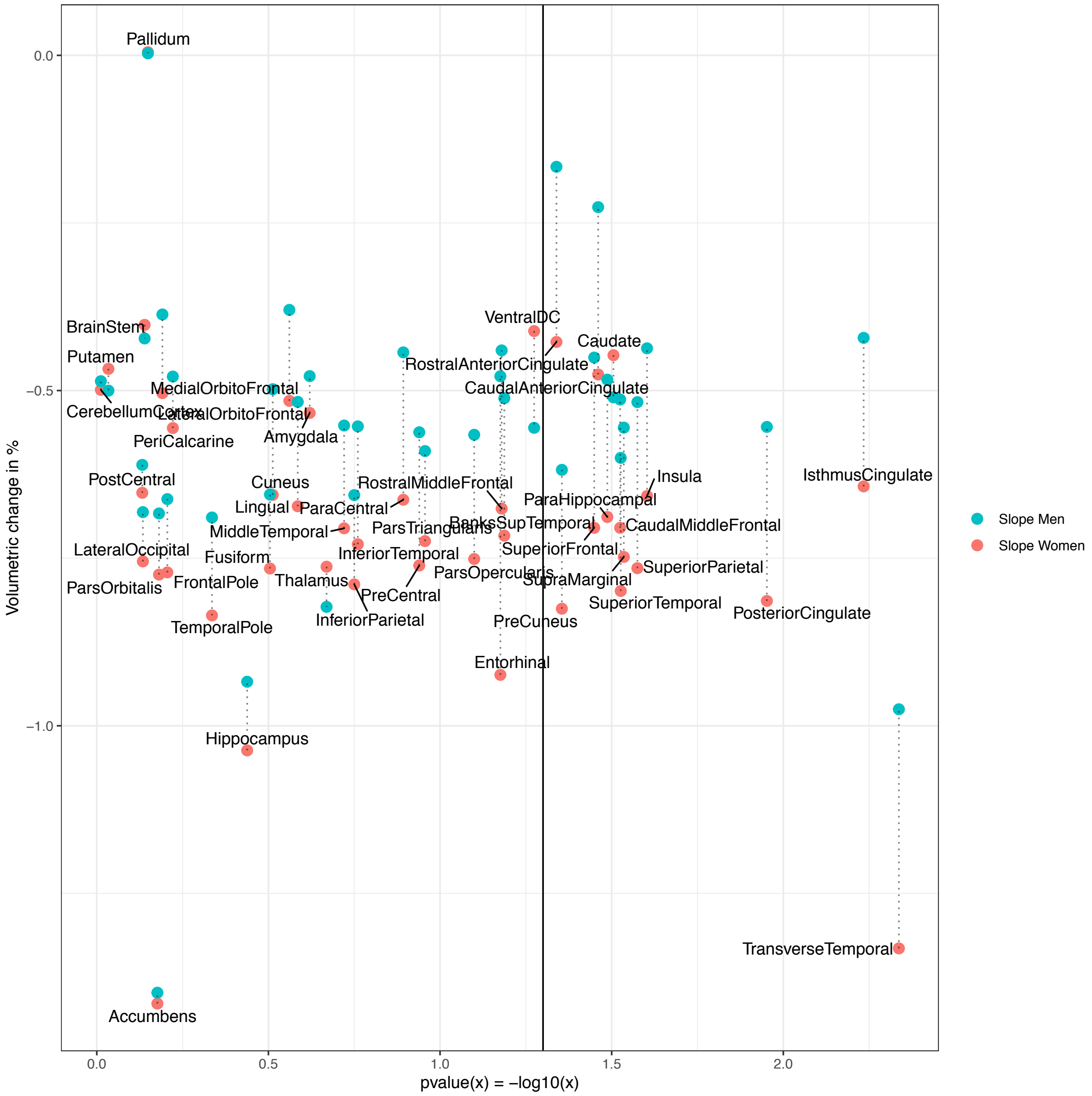

Figure S4: Expected slopes at an age of 70 years separated for men and women conditioned on the mean intracranial volume of the entire sample. The x-axis represents the -log10 transformed p-value obtained with a likelihood ratio test comparing a model including a separate slope and a separate slope x age-interaction parameter for men and women to a model without separate parameters. On the transformed scale a value of 1.3 corresponds to a p-value of 0.05 (indicated by the vertical line).

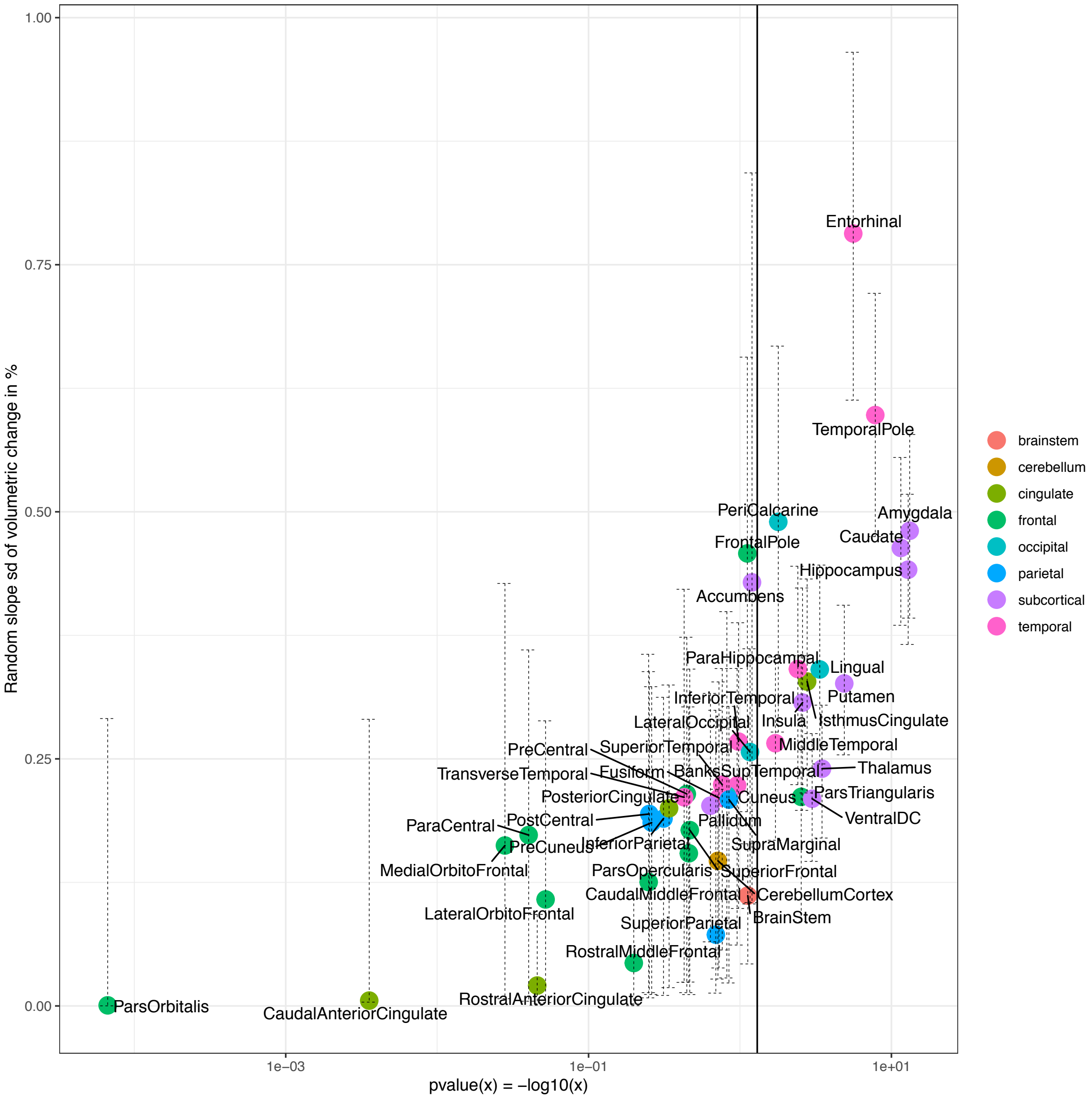

Figure S5: Random slope variance parameter. The x-axis represents the -log10 transformed p-value on the log10 scale obtained with a likelihood ratio test comparing a random slope model (with a covariance of random slope to random intercept) to a model containing only a random intercept. Values right to the vertical line correspond to p-values < 0.05. P-values > 0.05 indicate that the within-subject error was rather large compared to the random slope variation.

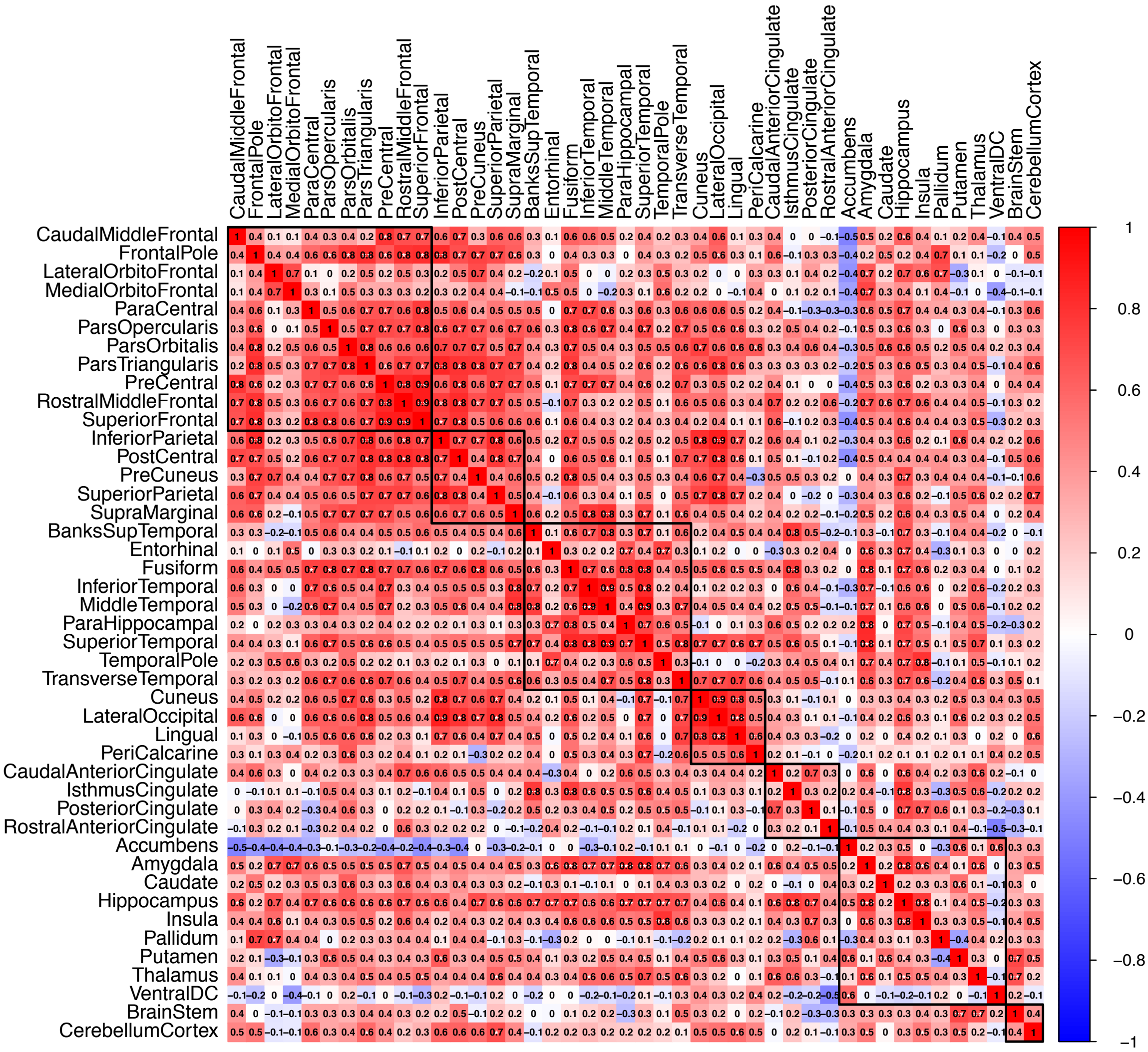

Figure S6: Medians of posterior distributions of correlations between the random slopes of different brain structures. * corresponds to a posterior mass < 0.05 on the other side of zero, ** corresponds to < 0.005, *** corresponds to < 0.0005.

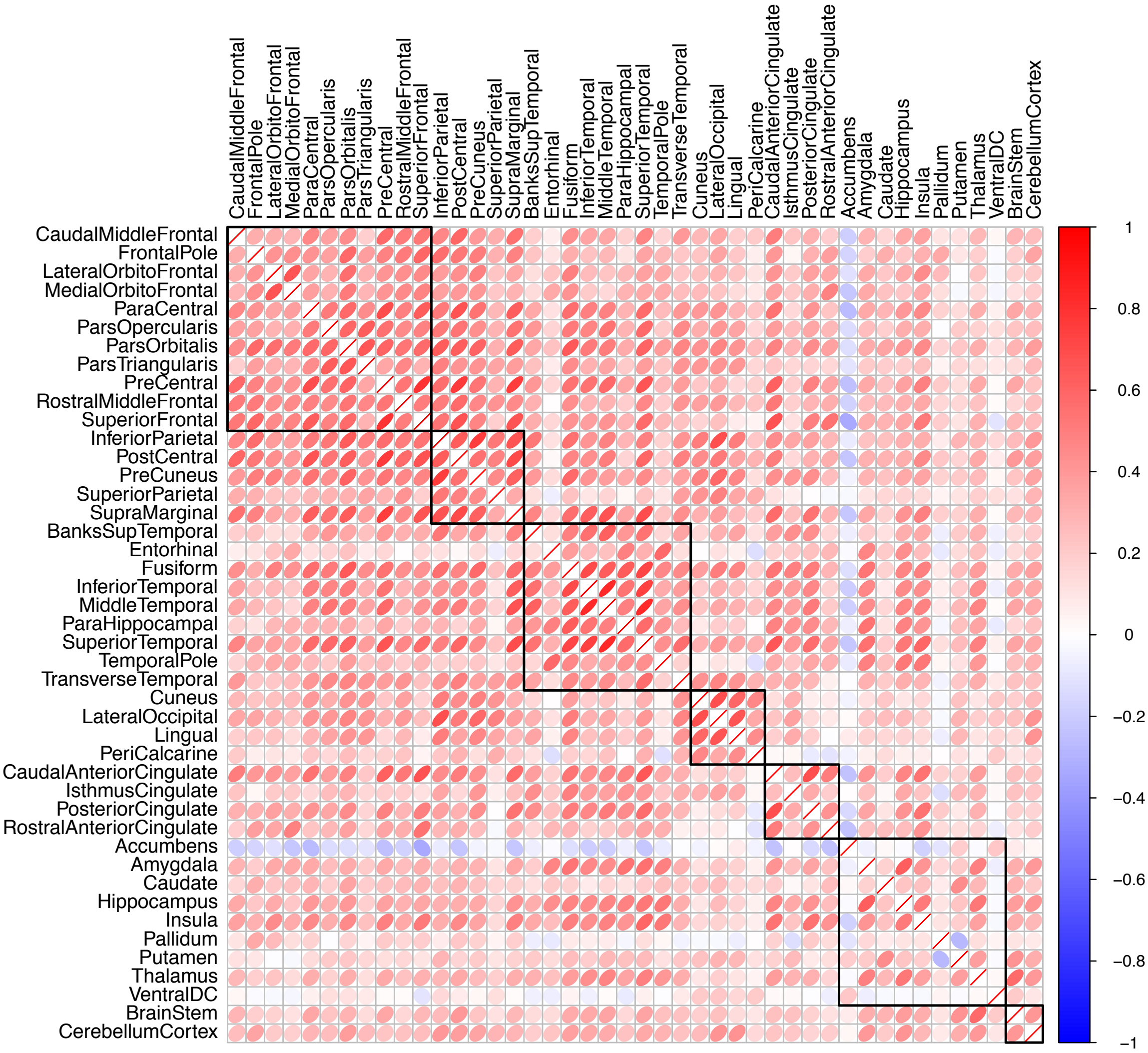

Figure S7: Simple estimate of multivariate correlation between the slopes of different brain structures obtained by using estimated factor scores. On these factor scores it was possible to do principle component analysis.

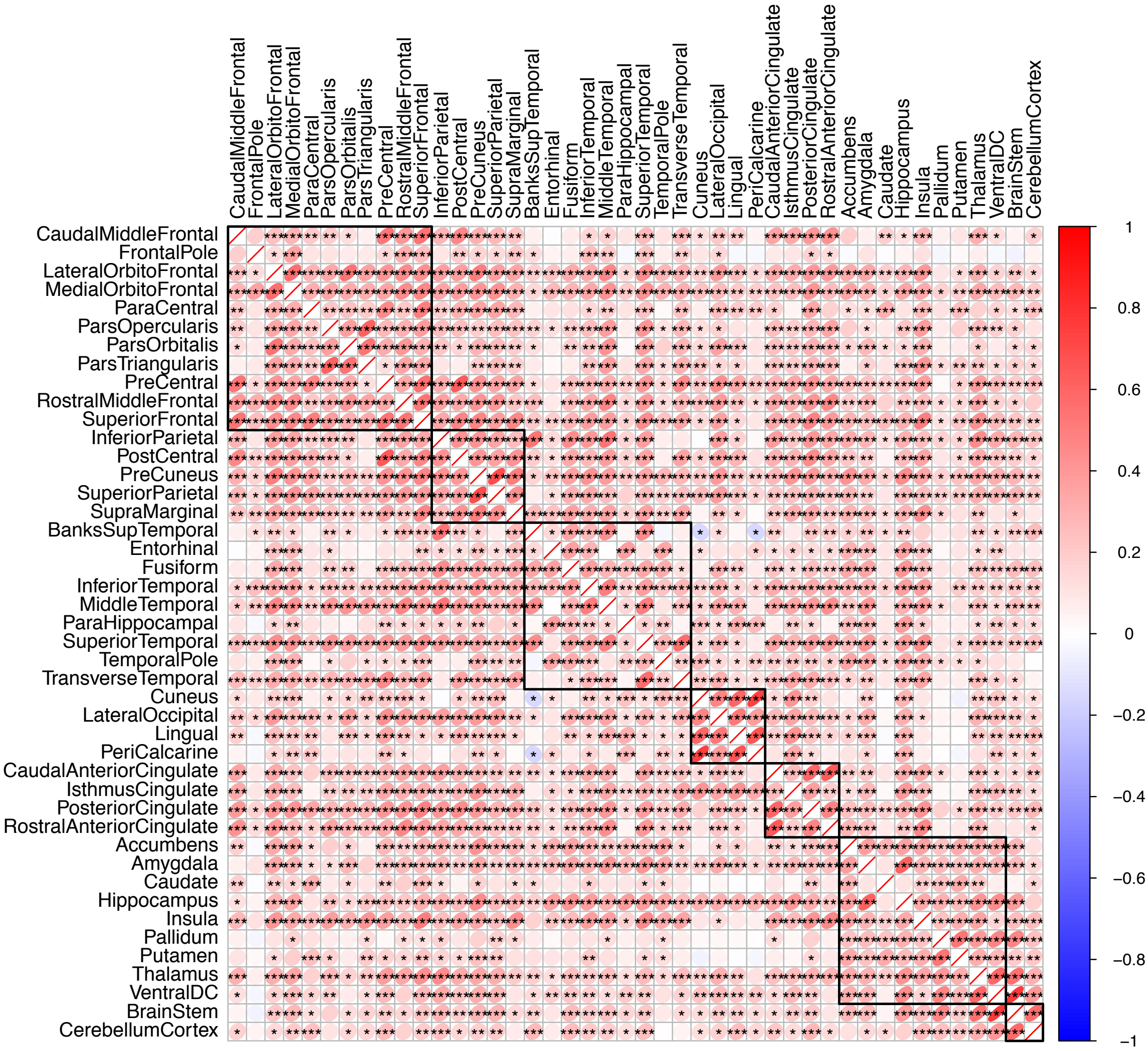

Figure S8: Level-level associations between brain structures. The medians of the posterior distributions of correlations between the random intercepts were obtained from the bivariate models. * corresponds to a posterior mass < 0.05 on the other side of zero, ** corresponds to < 0.005, *** corresponds to < 0.0005.

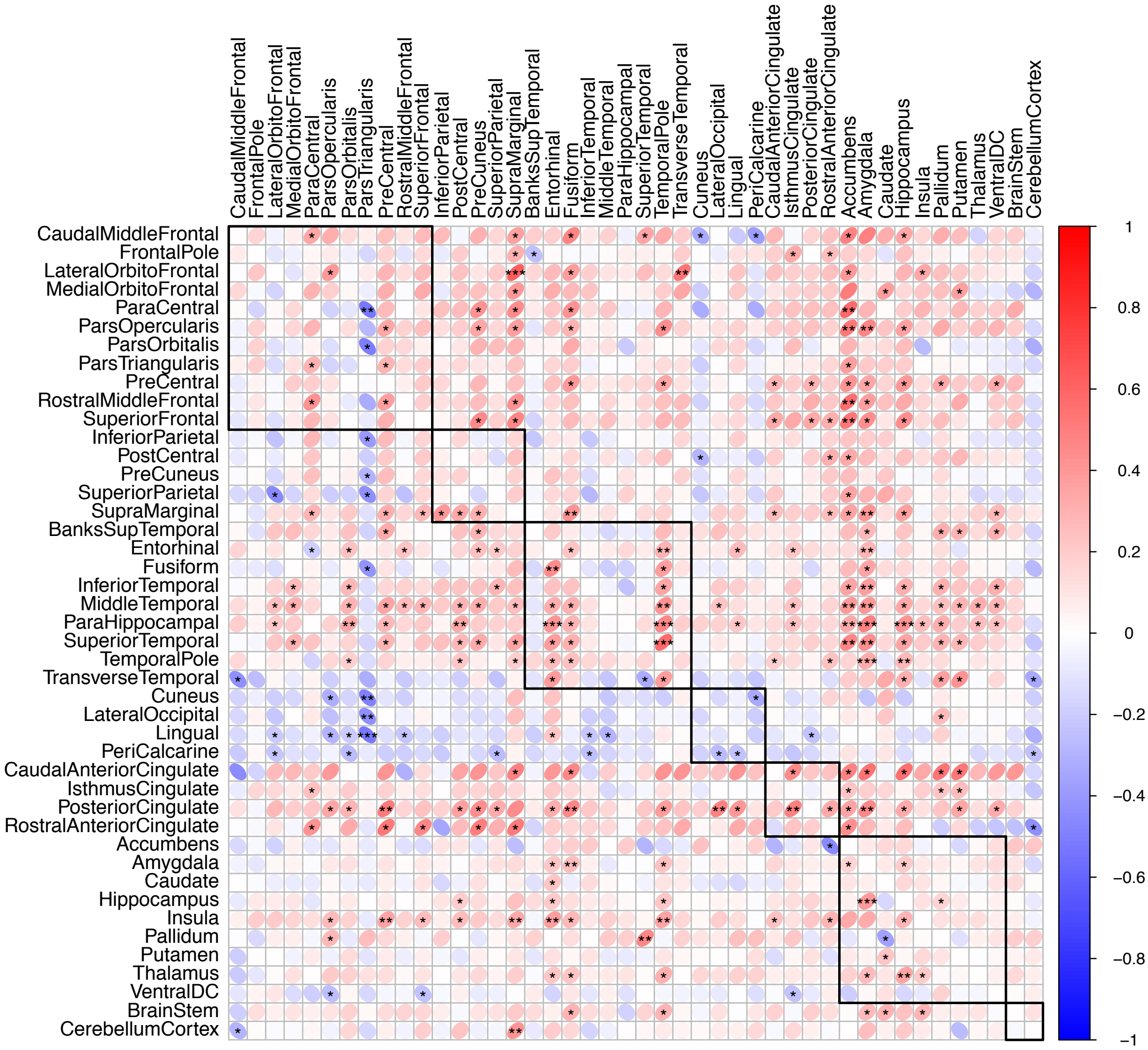

Figure S9: Level(rows)-change(columns) associations between brain structures. The medians of posterior distributions of correlation between random intercepts and random slopes were obtained from the bivariate models. * corresponds to a posterior mass $<$ 0.05 on the other side of zero, ** corresponds to <0.005, *** corresponds to <0.0005.
